# Mutations of MAP1B encoding a microtubule-associated phosphoprotein cause sensorineural hearing loss

**DOI:** 10.1172/jci.insight.136046

**Published:** 2020-12-03

**Authors:** Limei Cui, Jing Zheng, Qiong Zhao, Jia-Rong Chen, Hanqing Liu, Guanghua Peng, Yue Wu, Chao Chen, Qiufen He, Haosong Shi, Shankai Yin, Rick A. Friedman, Ye Chen, Min-Xin Guan

**Affiliations:** 1 Division of Medical Genetics and Genomics, The Children’s Hospital,; 2 Institute of Genetics and; 3 Department of Human Genetics, Zhejiang University School of Medicine, Hangzhou, Zhejiang, China.; 4 Deaprtment of Otorhinolaryngology, the Affiliated Hospital, Hangzhou Normal University, Hangzhou, Zhejiang, China.; 5 Department of Otorhinolaryngology, Shanghai Jiao Tong University Affiliated Sixth People’s Hospital, Shanghai, China.; 6 Division of Otolaryngology, University of California at San Diego School of Medicine, La Jolla California, USA.; 7 Zhejiang Provincial Key Laboratory of Genetic and Developmental Disorders, Hangzhou, Zhejiang, China.; 8 Joint Institute of Genetics and Genomic Medicine between Zhejiang University and University of Toronto, Zhejiang University, Hangzhou, Zhejiang, China.

**Keywords:** Genetics, Otology, Genetic diseases, Molecular pathology, iPS cells

## Abstract

The pathophysiology underlying spiral ganglion cell defect–induced deafness remains elusive. Using the whole exome sequencing approach, in combination with functional assays and a mouse disease model, we identified the potentially novel deafness-causative *MAP1B* gene encoding a highly conserved microtubule-associated protein. Three novel heterozygous *MAP1B* mutations (c.4198A>G, p.1400S>G; c.2768T>C, p.923I>T; c.5512T>C, p.1838F>L) were cosegregated with autosomal dominant inheritance of nonsyndromic sensorineural hearing loss in 3 unrelated Chinese families. Here, we show that *MAP1B* is highly expressed in the spiral ganglion neurons in the mouse cochlea. Using otic sensory neuron–like cells, generated by pluripotent stem cells from patients carrying the *MAP1B* mutation and control subject, we demonstrated that the p.1400S>G mutation caused the reduced levels and deficient phosphorylation of MAP1B, which are involved in the microtubule stability and dynamics. Strikingly, otic sensory neuron–like cells exhibited disturbed dynamics of microtubules, axonal elongation, and defects in electrophysiological properties. Dysfunctions of these derived otic sensory neuron–like cells were rescued by genetically correcting *MAP1B* mutation using CRISPR/Cas9 technology. Involvement of MAP1B in hearing was confirmed by audiometric evaluation of *Map1b* heterozygous KO mice. These mutant mice displayed late-onset progressive sensorineural hearing loss that was more pronounced in the high frequencies. The spiral ganglion neurons isolated from *Map1b* mutant mice exhibited the deficient phosphorylation and disturbed dynamics of microtubules. *Map1b* deficiency yielded defects in the morphology and electrophysiology of spiral ganglion neurons, but it did not affect the morphologies of cochlea in mice. Therefore, our data demonstrate that dysfunctions of spiral ganglion neurons induced by MAP1B deficiency caused hearing loss.

## Abstract 2

Dysfunctions of spiral ganglion neurons caused by Map1b deficiency leads to sensorineural hearing loss.

## Graphical abstract

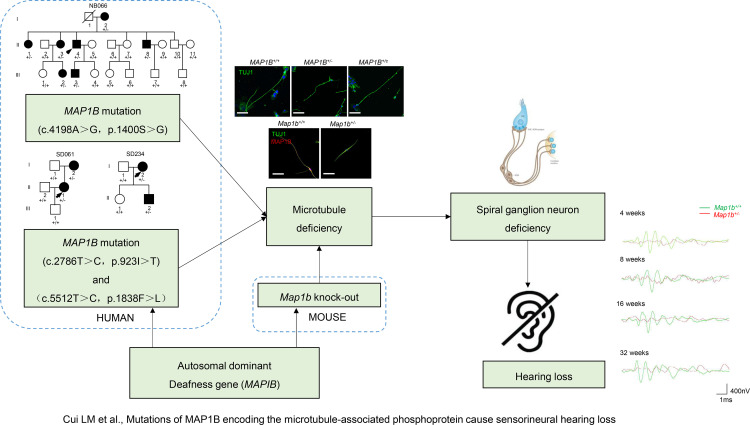

## Introduction

Hearing loss is the most common sensory disorder, affecting 500 million people worldwide (1, 2). Hearing loss can be grouped into syndromic hearing loss (hearing loss with other medical problems such as diabetes) and nonsyndromic hearing loss (hearing loss is the only obvious medical problem). Sensorineural hearing loss often occurs as a consequence of damaged or deficient cochlear hair cells. Indeed, the spiral ganglion cells serve the sense of hearing by sending a representation of sound from the cochlea to the brain. Hearing loss can be caused by gene alterations and environmental factors, including ototoxic drugs such as aminoglycoside antibiotics (3–5). Approximately 70% of subjects with nonsyndromic hearing loss have a genetic predisposition, with autosomal dominant (DNFA), autosomal recessive (DNFB), X-linked (DNFX), or maternally transmitted patterns of inheritance (6). Of these, autosomal dominant nonsyndromic hearing loss accounts for 20% of nonsyndromic hearing loss. In contrast with autosomal recessive form, autosomal dominant forms were often characterized by hearing loss with postlingual onset ranging from 10 to 50 years old, a progressive course and often a milder degree of hearing loss (6–8). Recent advances in genome sequence technology identified 37 genes linked to autosomal dominant hearing loss (http://hereditaryhearingloss.org). These genes are involved in the structure, development, and physiology of inner ear in many ways, including cytoskeleton of hair cells, hair cell adhesion, intracellular transport, ion hemostasis, extracellular matrix, and transcription factors (9–14). However, molecular components of spiral ganglion neurons (SGNs) linked to hearing loss remain largely unknown.

Our recent studies demonstrated that mutations in *GJB2* and mitochondrial 12S rRNA genes are the important causes in a large cohort of Chinese patients with nonsyndromic hearing loss (15, 16). In the present investigation, using whole exome sequencing (WES) of 863 genetically uncharacterized Chinese individuals, we identified 3 potentially novel variants (c.4198A>G, p.1400S>G; c.2768T>C, p.923I>T; c.5512T>C, p.1838F>L) in the *MAP1B* gene encoding a highly conserved microtubule-associated protein in 3 genetically unrelated Chinese pedigrees. The p.1400S>G, p.923I>T, and p.1838F>L mutations resided at highly conserved residues of MAP1B, which is involved in microtubule dynamics in growing axons and growth cones (17–20). In particular, Ser1400 in the MTA domain of MAP1B is located at a highly conserved phosphorylated site essential for the function of embryonic cortical neurons (21). It was therefore hypothesized that the substitution of Ser1400 with glycine resulted in the deficient phosphorylation of MAP1B and consequently led to dysfunction of otic neurons. To elucidate the pathophysiology of *MAP1B* mutation, we generated the induced pluripotent stem cell (iPSC) from the members of 1 Han Chinese family carrying the *MAP1B* p.1400S>G mutation and control subject and, subsequently, otic sensory neuron–like (OSN-like) cells differentiated from those iPSCs. These otic neuron-like cells were assessed for the effects of *MAP1B* p.1400S>G mutation on the phosphorylation activity, morphology, and electrophysiological properties. We then investigated if these defects in the cells can be rescued by CRISPR/Cas9-mediated gene correction. To examine whether defects in *MAP1B* cause the hearing dysfunction in vivo, we studied the *MAP1B* heterozygous KO mice produced by the genomic editing using the CRISPR/Cas9 system. In this manuscript, we demonstrate that *MAP1B*–KO mice recapitulated the abnormal morphology and dysfunction of patient derived-otic neuron-like cells and hearing-impaired phenotypes in patients.

## Results

### Identification of MAP1B mutations by WES.

**Figure 1:**
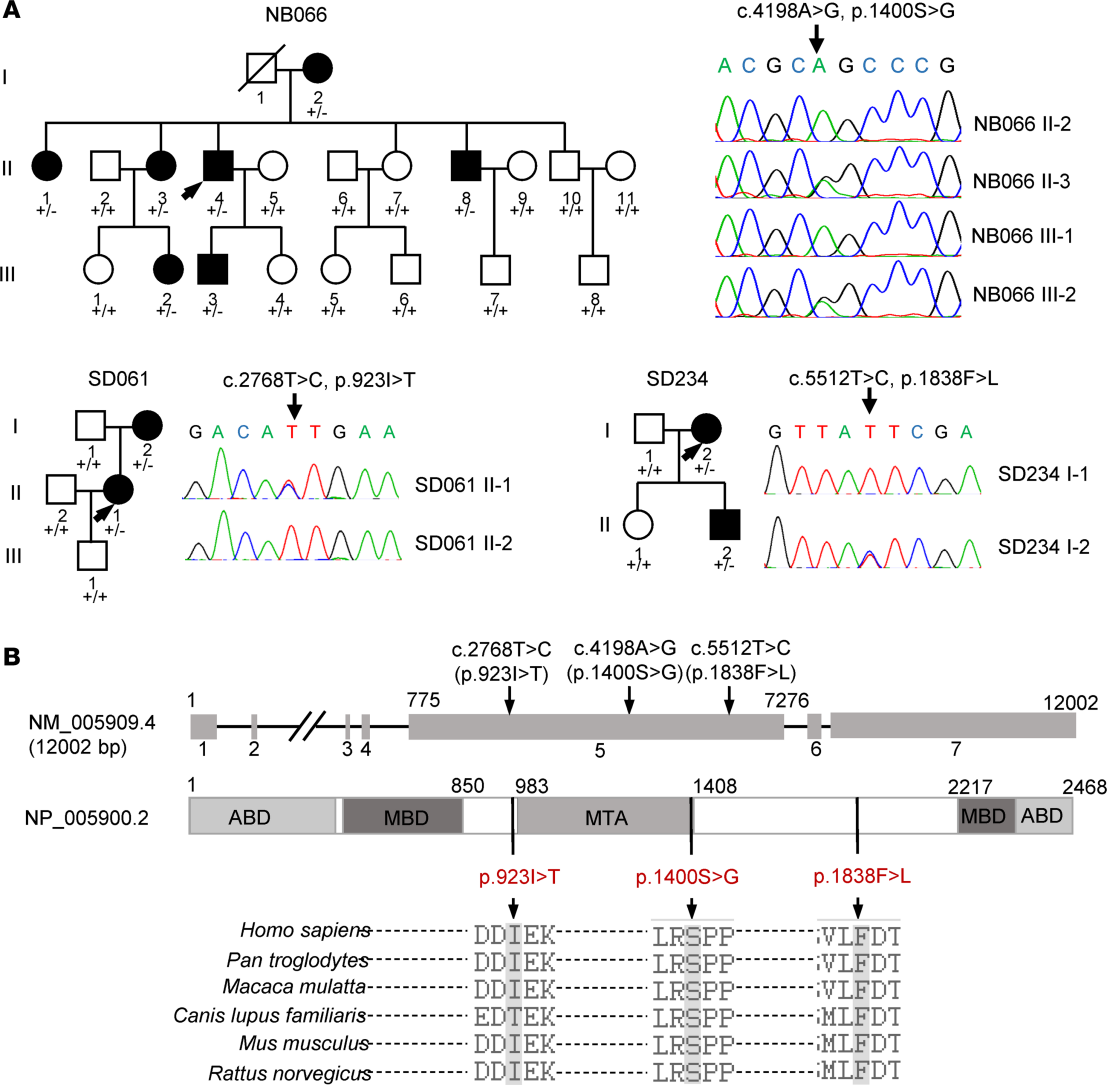
Identification of *MAP1B* mutations. (**A**) Three Han Chinese pedigrees with hearing loss and partial Sanger sequence chromatograms of *MAP1B* genes in some members. Hearing-impaired individuals were indicated by blackened symbols. Individuals harboring heterozygous (+/–) or WT (+/+) *MAP1B* mutations are indicated. (**B**) Scheme for the structure of human MAP1B and multiple sequence alignments of its homologs. Positions of p.923I>T, p.1400S>G, and p.1838F>L mutations were marked with arrows. ABD, actin binding domain; MBD, microtubule binding domain; MTA, putative microtubule assembly helping domain.

The Han Chinese family (NB066) used for this investigation was recruited from the ENT clinic of Zhejiang University School of Medicine. As shown in Figure 1A, this family exhibited the autosomal dominant inheritance of hearing loss. Seven of 21 members in this family exhibited bilateral sensorineural hearing impairment as a sole clinical phenotype. Members of this family displayed the late-onset/progressive hearing impairment with variable severity and age at onset (Supplemental Figure 1 and Supplemental Table 1; supplemental material available online with this article; https://doi.org/10.1172/jci.insight.136046DS1). As shown in Supplemental Figure 2, there were no significant difference of distortion product otoacoustic emissions (DPOAE) measurements between affected subjects and hearing-normal controls. These suggested that the hearing impairment of these affected members may result from the dysfunctions in the spiral ganglia neurons. Furthermore, none of the patients had any history of constant exposures to ototoxic drugs or noise.

Initial targeting exome sequencing analyses of 89 reported deafness-associated genes failed to identify any mutations (16). We then subjected genomic DNA from 2 hearing-impaired family members (II-3 and II-4) to WES. The overview of the exome analysis was summarized in Supplemental Figure 3. After removing annotated polymorphisms and filtering for variants, a single exonic variant (c.4198A>G, p.1400S>G) in the exon 5 of *MAP1B* (NC_000005.10) was identified in these 2 hearing-impaired individuals (Supplemental Table 2). The c.4198A>G mutation changed a highly conserved 1400 serine with glycine (p.Ser1400Gly) at the MTA domain of MAP1B, which is the highly conserved phosphorylated site essential for the function of embryonic cortical neurons (21). We then carried out the Sanger sequence analysis of DNA fragments spanning all exons and their flanking sequences of *MAP1B* among 7 affected patients and 13 unaffected members of this Chinese family. As shown in Figure 1A, this potentially novel mutation was present in all 7 affected patients but not in the 13 unaffected family members. No other sequence changes were detected among these individuals. We further analyzed the presence of the *MAP1B* c.4198A>G mutation in a cohort of 863 genetically unrelated hearing-impaired probands and 206 unrelated hearing-normal individuals by Sanger sequencing. We failed to detect the c.4198A>G mutation in all these hearing-normal and hearing-impaired individuals.

We then performed the Sanger sequence of DNA fragments spanning all exons and their flanking sequences of *MAP1B* in the 863 genetically unrelated hearing-impaired probands. Two potentially novel variants (c.2768T>C, p.923I>T; c.5512T>C, p.1838F>L) were identified in the heterozygous form in the hearing-impaired individuals (I-2, II-2) of SD061 pedigree and hearing-impaired members (I-2, II-2) of the SD234 pedigree, respectively. However, these 2 variants were absent in the asymptomatic individuals of these families and 206 hearing normal subjects. As shown in Figure 1B, the isoleucine 923, Ser1400, and phenylalanine 1838 in the MAP1B protein are highly conserved residues among *Homo sapiens*, *Pan troglodytes*, *Macaca mulatta*, *Canis lupus familiaris*, *Mus musculus*, and *Rattus norvegicus*. These variants were further evaluated by using ACMG laboratory guideline (22), as shown in Supplemental Table 3. Based on in silico predictions and the incidences of their variants in the general population, the p.1400S>G variant is very likely deleterious, while the p.923I>T and p.1838F>L variants need to be further explored. Therefore, the p.1400S>G mutation in the MAP1B was used for functional characterization as below.

### Generation of otic sensory neuron–like cells derived from iPSCs.

**Figure 2:**
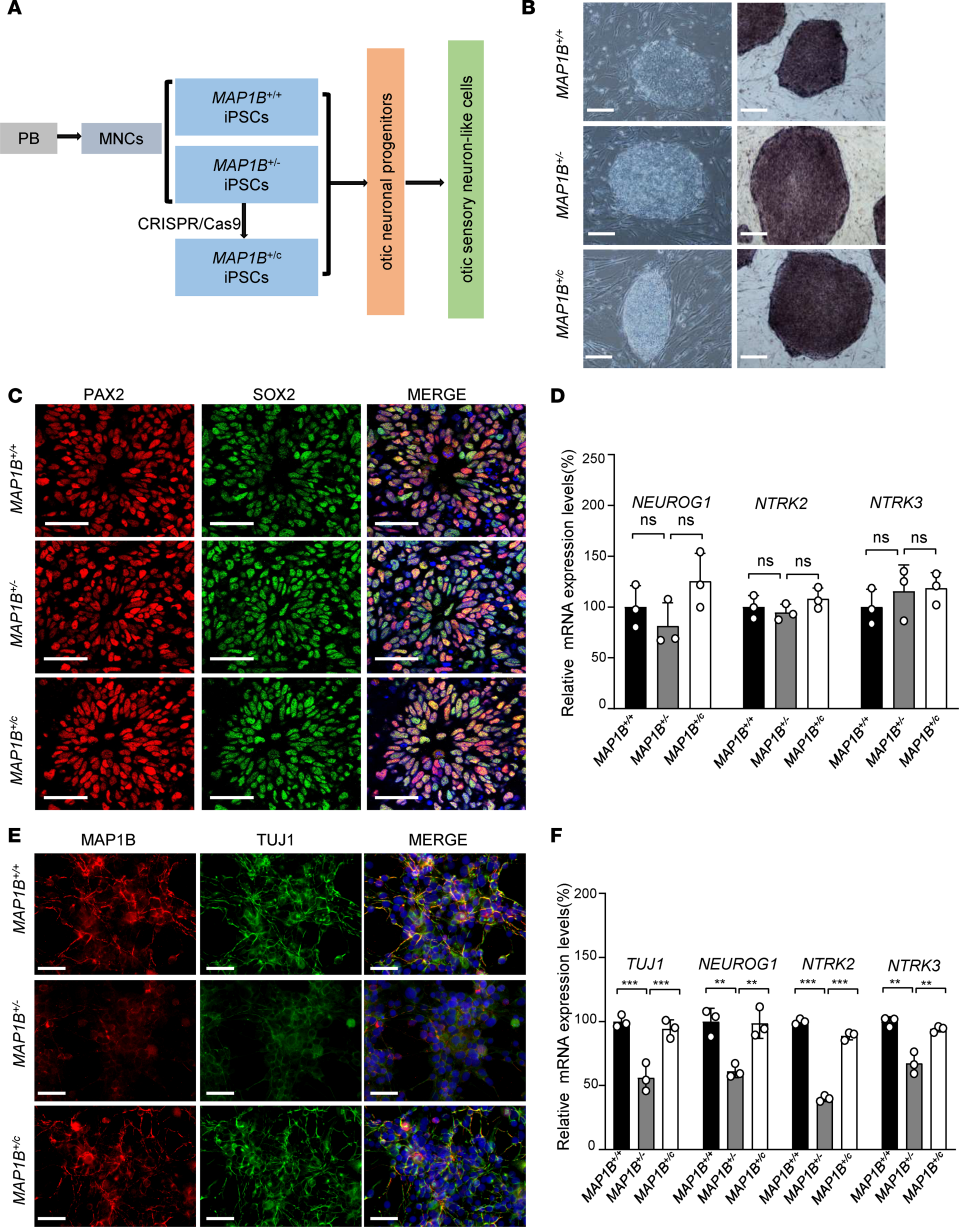
Generation of otic sensory neuron–like cells from patient-derived iPSCs. (**A**) Schematic diagram illustrating the neuronal differentiation processing from iPSCs with 3 different genotypes. Cells were derived from married-in control (NB066–II-6) and hearing-impaired subject (NB066–II-4), and these underwent CRISPR/Cas9-based genetic correction, presented as *MAP1B*^+/+^, *MAP1B*^+/–^, and *MAP1B*^+/c^, respectively. (**B**) Phase contrast microscopy and AP staining of the iPSC colonies. Scale bars: 200 μm. (**C**) iPSCs were induced toward otic neuronal progenitors and stained for the markers PAX2 (red) and SOX2 (green). Nuclei were stained with DAPI (blue). Scale bars: 40 μm. (**D**) *MAP1B, NEUROG1, NTRK2*, and *NTRK3* mRNA expression levels were analyzed using SYBR green real-time PCR and were normalized to *β*-actin mRNA expression. (**E**) Generations of otic neurons were confirmed by immunostaining with neuronal marker TUJ1 (green). Nuclei were stained with DAPI (blue). Scale bars: 50 μm. (**F**) *TUJ1*, *NEUROG1*, *NTRK2*, and *NTRK3* mRNA expression levels were analyzed in differentiated OSN-like cells. Data are mean ± SEM of triplicates. *P < 0.05 and **P <0.01 by 1-way ANOVA followed unpaired Student’s *t* test.

It is necessary to establish the direct functional link between the p.1400S>G mutation and dysfunction of otic neurons. In fact, *MAP1B* is expressed at high levels in the neuron system but extremely low levels in other tissues (23, 24). These led us to generate iPSCs from peripheral blood mononuclear cells (PBMNCs) donated by both hearing-impaired subjects and married-in, hearing-normal members in this pedigree, and to differentiate these iPSCs into OSN-like cells for morphological and physiological characterizations. As illustrated in Figure 2A, 2 iPSC lines were generated from the PBMNCs of the proband II-4 (*MAP1B^+/–^* iPSCs) and married-in control II-6 (*MAP1B^+/+^* iPSCs) by electroporation expression vectors bearing reprogramming factors. To investigate if the genetic correction of MAP1B p.1400S>G mutation can rescue the physiological phenotype, the p.1400S>G mutation in iPSC lines derived from the proband II-4 was corrected by CRISPR/Cas9, and the resultant PSCs were referred as *MAP1B^+/c^* iPSCs (Supplemental Figure 4A). Sanger sequencing results confirmed the correction of the c.4198A>G allele and no off-targeting sequence (Supplemental Figure 4, B and C). All iPSCs exhibited the morphological characteristics of human ESCs. They displayed staining positive for alkaline phosphatase (Figure 2B) and expression of pluripotent markers, including NANOG, OCT4, TRA1-60, SOX2, and KLF4 (Supplemental Figure 5, A and B). Embryoid bodies (EBs) and teratomas were formed, and both of them differentiated into cells of all 3 germ layers (Supplemental Figure 5, C and D). These results indicated that we successfully generated 3 iPSC lines (*MAP1B^+/+^* iPSCs, *MAP1B^+/–^* iPSCs, and *MAP1B^+/c^* iPSCs).

The iPSCs underwent directed differentiated into OSN-like cells using a stepwise differentiation procedure as shown in Figure 2A. To determine whether the cells had started differentiating toward early otic neuronal lineage, we assessed the dynamic expression of a panel of lineage markers expressing during otic/placodal development. In particular, we used a combined expression of PAX2 plus SOX2 and PAX2 plus NESTIN to track the differentiation of iPSCs with *MAP1B^+/+^*, *MAP1B^+/–^*, and *MAP1B^+/c^* genotypes toward otic neuronal progenitor cells (ONPs) (25–27). Immunocytochemistry analyses showed that differentiated cells from iPSCs started expressing *PAX2*, *SOX2*, and *NESTIN* at day 15 and ~85% double-positive cells in all 3 groups (Figure 2C and Supplemental Figure 6A). The reverse transcription PCR (RT-PCR) analyses revealed the expression of neurosensory progenitor markers (*PAX2*, *SOX2, EYA1, SIX1*) and OSN markers (*NEUROG1, NTRK2*, and *NTRK3*) in 3 genotype iPSCs-derived ONPs but no significant differences of their levels among the 3 groups (Figure 2D and Supplemental Figure 6B) (28–30). The subsets of PAX2^hi^SOX2^hi^Nestin^hi^-expressing cells representing the otic progenitors were further determined by flow cytometry.

These otic progenitor cells induced OSN differentiation in a neurobasal medium. After growing 20 days, differentiated cells with typical neuron morphology (with cell body and neurites) in all 3 groups were shown by immunocytochemical stains with otic neuronal markers TUJ1, NF-H, and VHLUT1 (Figure 2E and Supplemental Figure 6C) (31). Notably, marked reductions in the fluorescence densities of MAP1B, TUJ1, NF-H, and VHLUT1 were observed in *MAP1B^+/–^* cells, as compared with those in *MAP1B^+/+^* cells and restored in *MAP1B^+/c^* cells (Figure 2E and Supplemental Figure 6C). The quantitative PCR (qPCR) analysis displayed the significant decreases in the levels of *MAP1B*, *NEUROG1, NTRK2*, and *NTRK3* mRNA in OSN-like cells with *MAP1B^+/–^* cells, as compared with those in the *MAP1B^+/+^* cells (Figure 2F and Supplemental Figure 6D). These differentiated cells (TUJ1^+^ staining) were further microphotographed using a FV1000 confocal laser-scanning microscope to examine if these cells contained neurites. Among 200 cells in each group, more than 95% of differentiated cells (TUJ1^+^) of *MAP1B*^+/+^ or MAP1B^+/c^ group contained at least 1 neurite, while only ~85% differentiated cells (TUJ1^+^) in *MAP1B*^+/–^ group had neurites.

To further evaluate the effect of *MAP1B* mutation on OSN differentiation, we performed qPCR analysis for *MAP1B*, *NEUROG1, NTRK2*, and *NTRK3* using total RNA isolated from iPSCs, ONPs, and OSN-like cells as templates. Through the induction of neuron differentiation, the levels of *MAP1B*, together with *NEUROG1, NTRK2*, and *NTRK3,* were markedly increased (Supplemental Figure 7). However, both *MAP1B^+/–^* and *MAP1B^+/+^* OSN-like cells exhibited an average ~2.4-fold increases in the levels of *NEUROG1*, *NTRK2*, and *NTRK3* mRNA, as compared with those in their iPSCs. These results suggested that the p.1400S>G mutation did not affect the differentiating process of iPSCs toward ONPs. Strikingly, 6.1- and 10.2-fold increases in the average levels of *NEUROG1, NTRK2*, and *NTRK3* were observed in the *MAP1B^+/–^* and *MAP1B^+/+^* OSN-like cells, as compared with those in their OSP-like cells, respectively. These data indicated that the p.1400S>G mutation led to a neuronal differentiation defect.

### The p.1400S>G mutation affected the MAP1B and neuron function.

**Figure 3:**
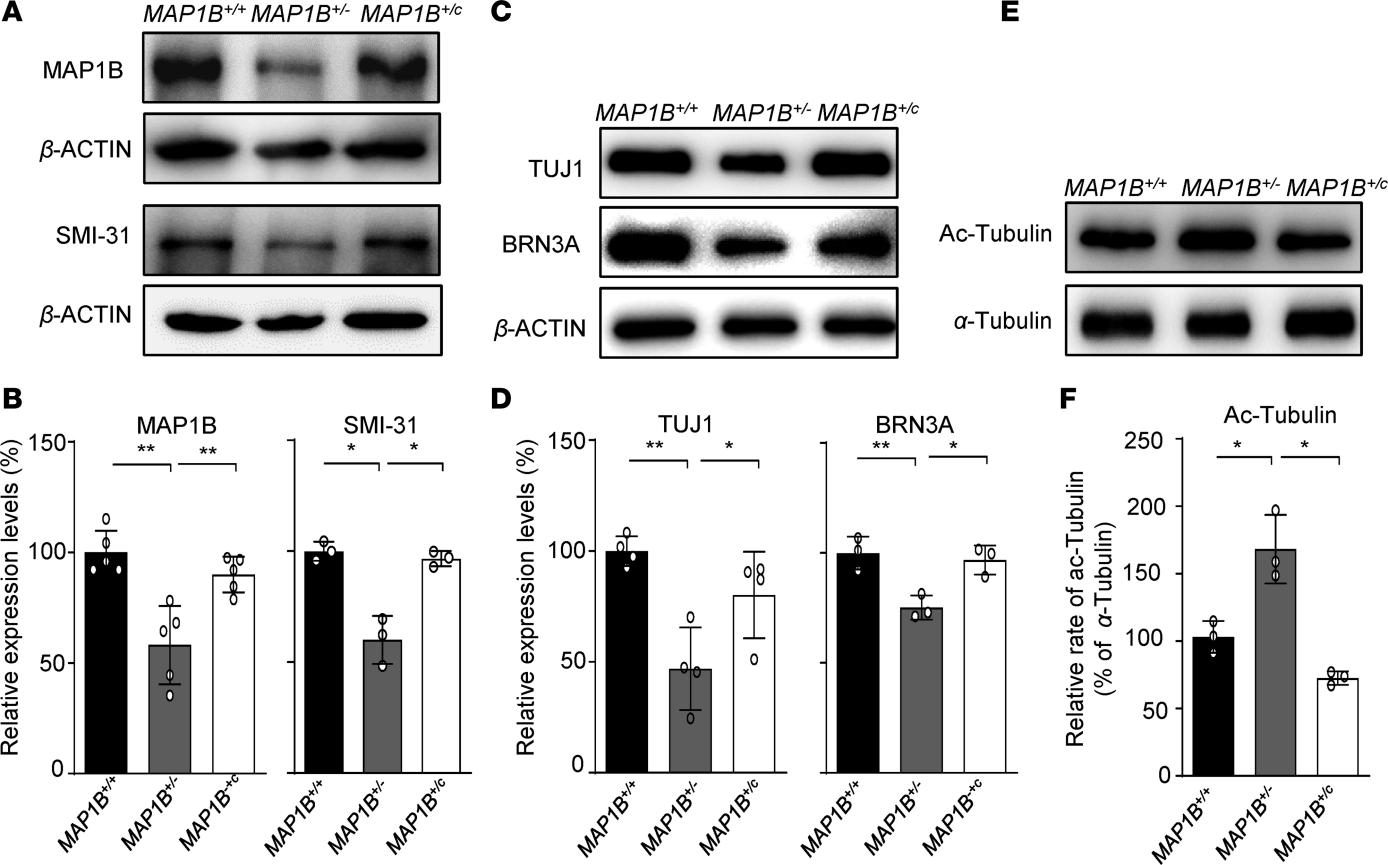
Protein expression analysis of OSN-like cells with 3 different genotypes. (**A** and **B**) Expression levels of MAP1B and SMI-31 in differentiated OSN-like cells with 3 different genotypes. A total of 15 μg of total cellular proteins of each cell line was electrophoresed through SDS-PAGE and incubated with MAP1B, SMI-31 antibodies, and β-actin as a loading control. Quantification of MAP1B and SMI-31 levels were determined by ImageJ software. The values for the mutant cell lines were expressed as percentages of the average values for the control cell lines. (**C** and **D**) Levels of neuronal marker TUJ1 and sensory neuron marker BRN3A in OSN-like cells with different genotypes. (**E**) Levels of α-tubulin and acetylated tubulin in 3 different genotypes of OSN-like cells by Western blotting analysis. (**F**) Relative levels of tubulin acetylation were quantified and normalized to *MAP1B*^+/+^ cells. Data are mean ± SEM of triplicates. *P <0.05 and **P <0.01 by 1-way ANOVA followed unpaired Student’s *t* test.

To experimentally test the predicted effect of p.1400S>G mutation on the MAP1B function, we examined the levels of MAP1B by the Western blotting analysis in these OSN-like cells with different genotypes (*MAP1B^+/–^*, *MAP1B^+/c^*, and *MAP1B*^+/+^). As shown in Figure 3, A and B, the average levels of MAP1B in the *MAP1B^+/–^* and *MAP1B^+/c^* cells were 63% and 90% of the average values of *MAP1B*^+/+^ cells after normalization with β-actin, respectively. Furthermore, the type I phosphorylation levels of MAP1B, detected by SMI-31 in the *MAP1B^+/–^* and *MAP1B^+/c^* cells, were 69% and 96% of those in the *MAP1B*^+/+^ cells (32, 33). These suggested that the p.1400S>G mutation caused the instability and deficient phosphorylation of MAP1B.

We assessed whether the p.1400S>G mutation affected the neuron function using neuronal marker TUJ1 and sensory neuron marker BRN3A antibodies (34). As illustrated in Figure 3, C and D, the levels of TUJ1 and BRN3A in the *MAP1B*^+/–^ OSN-like cells were 57% and 67% of those in the *MAP1B*^+/+^ cells, respectively. Strikingly, the levels of TUJ1 and BRN3A in the *MAP1B^+/c^* OSN-like cells were 80% and 96% of those in the *MAP1B*^+/+^ cells, respectively. These data indicate that the p.1400S>G mutation altered the neuron function, which was restored by the genetic correction of p.1400S>G mutation in the *MAP1B^+/c^* OSN-like cells.

The phosphorylation of MAP1B is required for maintaining the pool of dynamic microtubules (35). The acetylated α-tubulin is the marker for microtubule stabilization (36). We assessed if the p.1400S>G mutation affected the stability of microtubules by hybridizing Western blot using cellular proteins derived from these OSN-like cells with the acetylated-tubulin (ac-tubulin) antibody and α-tubulin as the internal control. As shown in Figure 3, E and F, the levels of acetylated-tubulin in the *MAP1B*^+/–^ and *MAP1B*^+/c^ cells were 168% and 75% of *MAP1B*^+/+^ cells, after normalization with α-tubulin, respectively. The increased levels of acetylated-tubulin implied that the p.1400S>G mutation caused the disturbing dynamics of microtubules.

### Impaired microtubule dynamics and axonal elongation.

**Figure 4:**
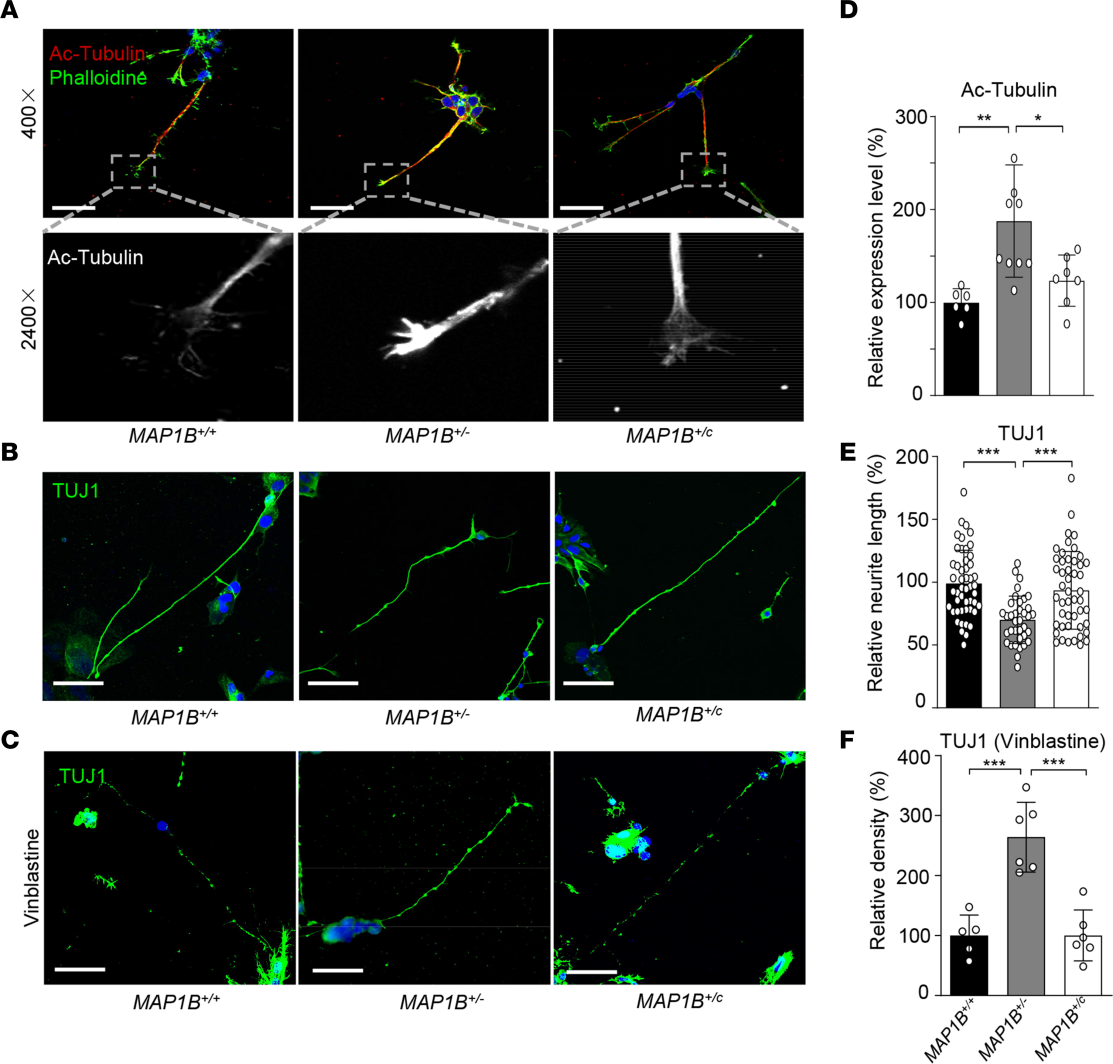
MAP1B p.1400S>G mutation altered axon elongation and microtubule stability in differentiated OSN-like cells. (**A** and **D**) Representative pictures of developing axons were costained with phalloidin (green) and an antibody against acetylated tubulin (red). Scale bars: 50 μm. (**B** and **E**) All 3 different genotypes of OSN-like cells showed long neurites and were positive for anti-TUJ1 staining (green). Scale bars: 50 μm. *n* = 40, 50, and 48 OSN-like cells for *MAP1B*^+/+^, *MAP1B*^+/–^, and *MAP1B*^+/c^ genotypes, respectively. (**C** and **F**) The differentiated OSN-like cells were treated with vinblastine and stained with TUJ1 (green). Scale bars: 50 μm. Nuclei were stained with DAPI (blue). Qualification of relative level of Ac-tubulin (**D**), relative neurite length (**E**), and relative density of TUJ1 (**F**) were shown. Data are mean ± SEM of triplicates. *P <0.05, **P <0.01, and ***P <0.001 by 1-way ANOVA followed unpaired Student’s *t* test.

The effect of the MAP1B p.1400S>G mutation on the dynamics and morphology of microtubules in the neurons was assessed by immunostaining analyses in OSN-like cells. First, we measured the level of acetylation of α-tubulin in OSN-like cells. As shown in Figure 4, A and D, marked increases in acetylation of α-tubulin were observed in the growth cones of developing axons carrying the p.1400S>G mutation, while the level of acetylation of α-tubulin in the *MAP1B*^+/c^ cells was comparable with those in *MAP1B*^+/+^ cells. Elevated levels of acetylated α-tubulin in these highly dynamic and motile structures at the leading edge of developing axons indicated a potential restriction of axon elongation.

We then measured the neurite lengths in differentiated OSN-like cells using immunocytochemical stain with TUJ1. Due to the variability of these cells, we selected differentiated cells with the most typical neuronal morphology in each group. As illustrated in Figure 4B, the neurite lengths in the *MAP1B*^+/–^differentiated neurons were markedly reduced, as compared with those in the *MAP1B*^+/+^ neurons. In particular, the average neurites lengths (140.3 ± 6.3 μm, *n* = 40) in the *MAP1B*^+/–^differentiated neurons were reduced 30% as compared with those (198 ± 7.6 μm, *n* = 50) in the *MAP1B*^+/+^ neurons. In contrast, the average neurite lengths (187.1 ± 8.9 μm, *n* = 48) in *MAP1B*^+/c^ neurons were comparable with those in the *MAP1B*^+/+^ neurons (Figure 4E).

Furthermore, we investigated whether the p.1400S>G mutation altered the microtubule polarization of these OSN-like cells after the administration of vinblastine, which can promote microtubule depolymerization (37). After the drug treatment, the levels of TUJ1 staining signals among *MAP1B*^+/+^, *MAP1B*^+/–^, and *MAP1B*^+/c^ OSN-like cells were decreased 80.4%, 48.2%, and 78.3%, respectively, as compared with corresponding cells without exposure to the drug (Figure 4, C and F), indicating that the cells carrying the p.1400S>G mutation were resistible to the depolymerizing drug treatment. These results indicated that the p.1400S>G mutation increased the stability of microtubules in the neurons and affected the microtubule dynamic states required for steering and extension.

### Defects in electrophysiological properties.

**Figure 5:**
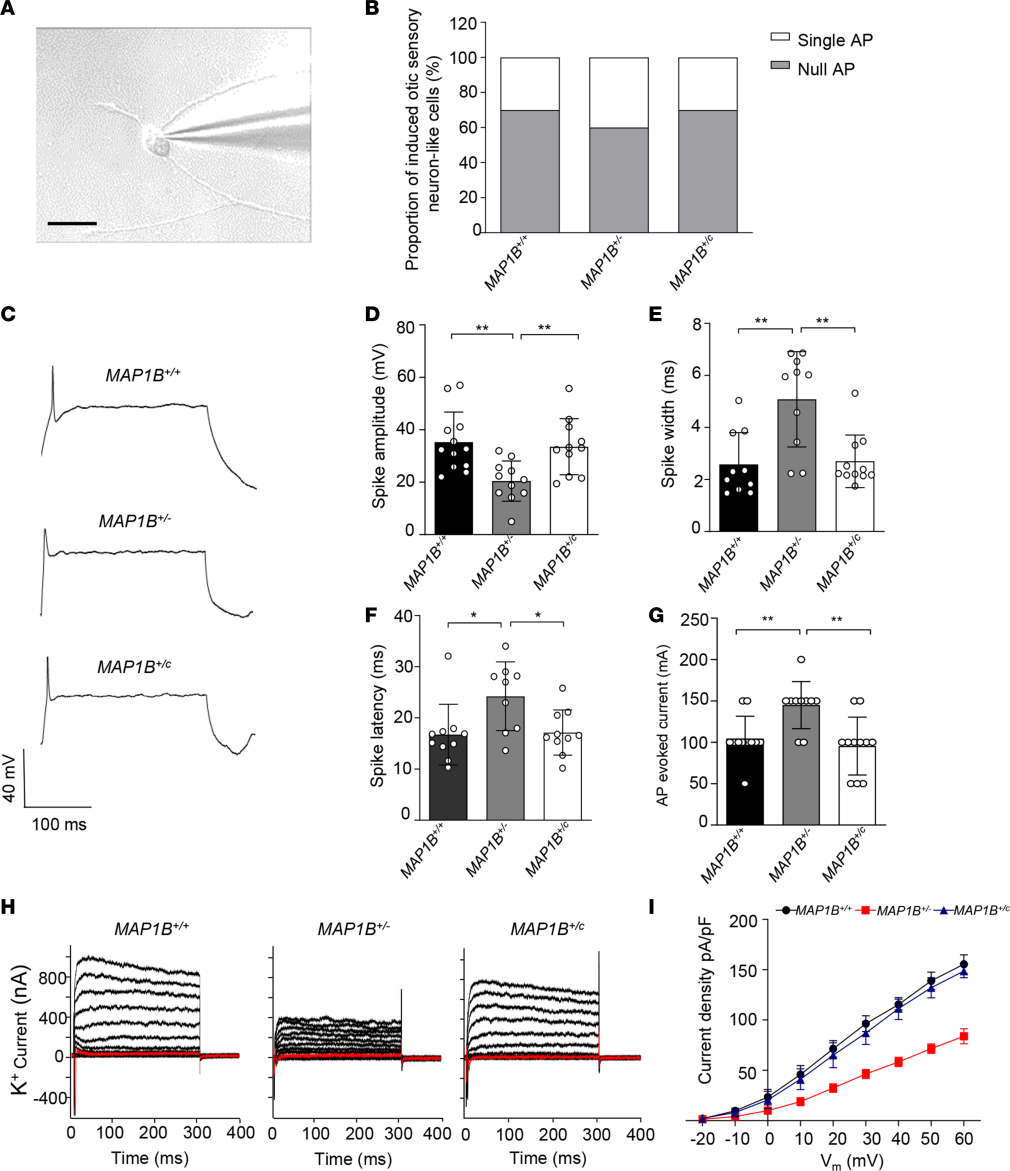
Patch-clamp analysis of OSN-like cells. (**A**) Photomicrograph showing a patch pipette attached to a differentiated OSN-like cell. Scale bar: 50 μm. (**B**) Current-clamp recording for *MAP1B*^+/+^, *MAP1B*^+/–^,and *MAP1B*^+/c^ OSN-like cells; proportion of 3 kinds of OSN-like cells showing null/single action potential firing as evoked with step-current injection. (**C**) Example recording of single action potential firing as evoked by current injection. (**D**–**G**) Spike amplitudes (**D**), spike widths (**E**), spike latencies (**F**), and thresholds of action-potential activation (**G**) were analyzed in 3 different OSN-like cells and normalized to *MAP1B*^+/+^ OSN-like cells. (**H**) Voltage-dependent K^+^ currents were recorded, stepping up from –80 mV to 60 mV in 10 mV increments. (**I**) Average peak current densities were calculated, and statistics were analyzed. Average peak current density indicates the peak current/the capacitance. Data are mean ± SEM of triplicates. *P <0.05 and **P <0.01 by 1-way ANOVA followed unpaired Student’s *t* test.

To examine the electrophysiological properties, differentiated cells with the typical neuronal morphology (cell body and neurites) in each genotype were selected and analyzed by whole-cell patch-clamp recordings. These cells displayed the bipolar morphology (Figure 5A). We recorded 20 cells that fired action potentials in response to depolarizing current pulses for each genotype. In fact, large proportions of mutant and WT OSN-like cells (70% of *MAP1B^+/+^*, 60% of *MAP1B^+/–^*, 70% of *MAP1B^+/c^*) fired a single spike in response to depolarization (Figure 5B). *MAP1B^+/–^* OSN-like cells exhibited significantly decreased spike amplitudes (42%), prolonged spike width (196.2%), longer spike latency (144.8%), and raised threshold of action-potential activation (139.4%), as compared with those of *MAP1B^+/+^* cells (Figure 5, C and D). In response to incremental voltage steps from –80 mV to +60 mV (10 mV increments), the OSN-like cells exhibited an outward rectifying K^+^ current. The sustained portion of the outward current increased steadily with increasing depolarization (Figure 5, D–G). Notably, the *I_K_* recorded in *MAP1B^+/–^* OSN-like cells were substantially lower than that in *MAP1B^+/+^* OSN-like cells (Figure 5, H and I). In contrast, those electrophysiological properties of *MAP1B^+/c^* OSN-like cells were comparable with those of *MAP1B^+/+^* OSN-like cells (Figure 5, C–I). These data demonstrate that the MAP1B p.1400S>G mutation impaired the membrane and electrophysiological properties, which are the characteristics of functional neurons, in the OSN-like cells. These dysfunctions were rescued by the genetic correction of the p.1400S>G mutation.

### Map1b is highly expressed in the SGNs.

**Figure 6:**
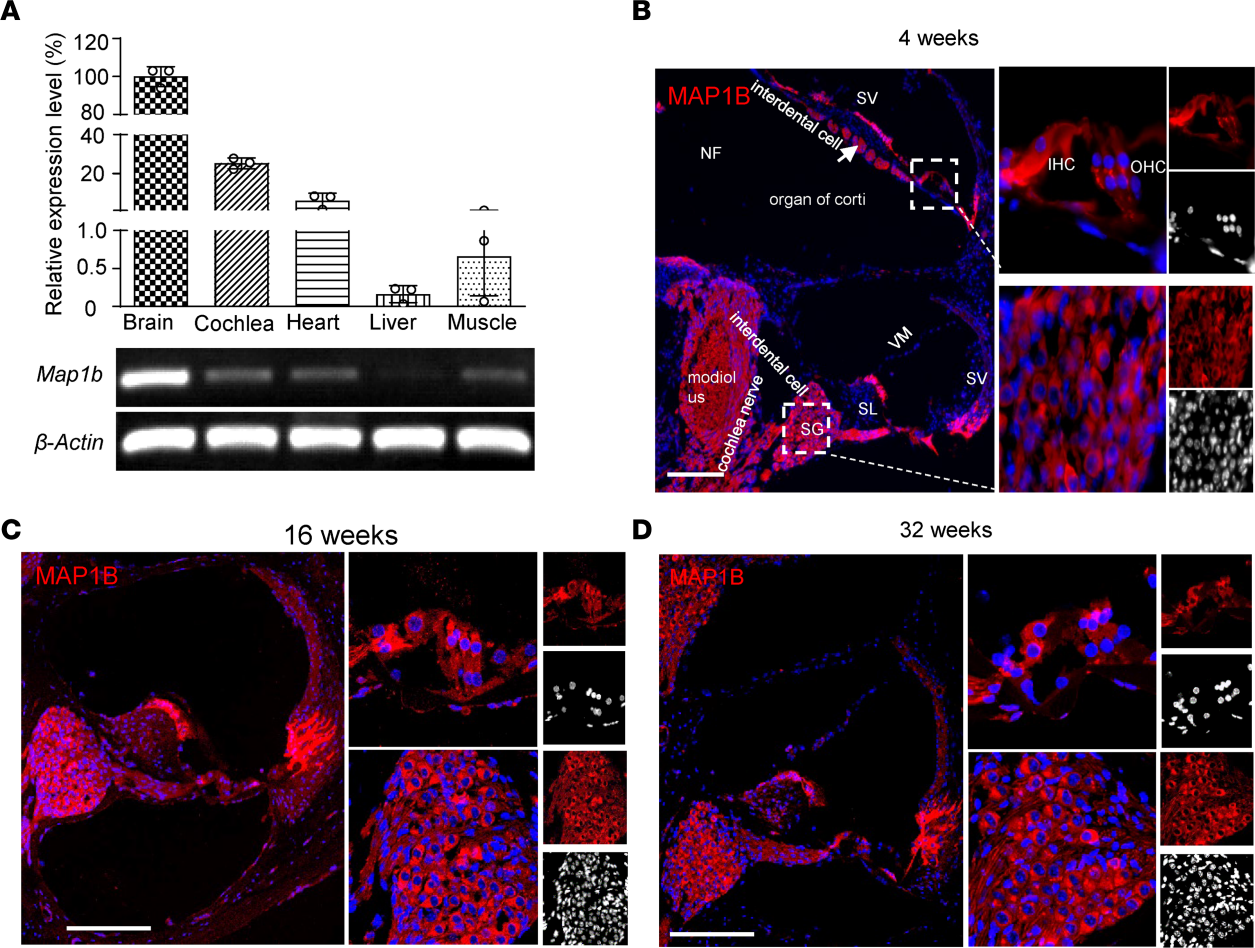
MAP1B is highly expressed in the spiral ganglion neurons. (**A**) Expression of *Map1b* among the different mouse tissues. Gene expression levels of *Map1b* were normalized to the average levels of β-actin in different tissues. (**B**–**D**) Immunofluorescence analysis of MAP1B expression in the mice cochlea and spiral ganglion at the age of 4 weeks (**B**), 16 weeks (**C**), and 32 weeks (**D**). Scale bars: 200 μm. SG, spiral ganglion; SL, spiral limbus; SV, stria vascularis; VM, vestibular membrane; OHC, outer hair cell; IHC, inner hair cell. Three independent experiments were performed.

To further investigate the role of *MAP1B* in the hearing function, we analyzed the expression patterns of *Map1b* in the mouse cochlea. Using RT-PCR analysis of *Map1b* in the brain, ear, heart, liver, and muscle of WT mice at the age of 4 weeks, we showed that *Map1b* was highly expressed in brain and cochlea, mildly expressed in heart, but very weakly expressed in liver and muscle (Figure 6A). We then examined the expression of *Map1b* in the various areas of cochlea, including spiral ganglion, spiral limbus, stria vascularis, vestibular membrane, outer hair cell, and inner hair cells. As shown in Figure 6B, immunofluorescence microscopy images of WT mice (at 4 weeks of age) cochlea revealed that *Map1b* was expressed in many areas in the cochlea, including the spiral ganglia neurons, mesenchymal cells, supporting cells, and even inner hair cells. Strikingly, *Map1b* is abundantly expressed in the spiral ganglions but is weak in hair cells. As shown in Figure 6, C and D, the expression levels and patterns of *Map1b* at various areas of the cochlea in the mice at ages of 16 and 32 weeks were comparable with those at the age of 4 weeks. These implicated that MAP1B played an important role in the function of spiral ganglia neurons.

### Map1b mutant mice exhibited a hearing-impaired phenotype.

**Figure 7:**
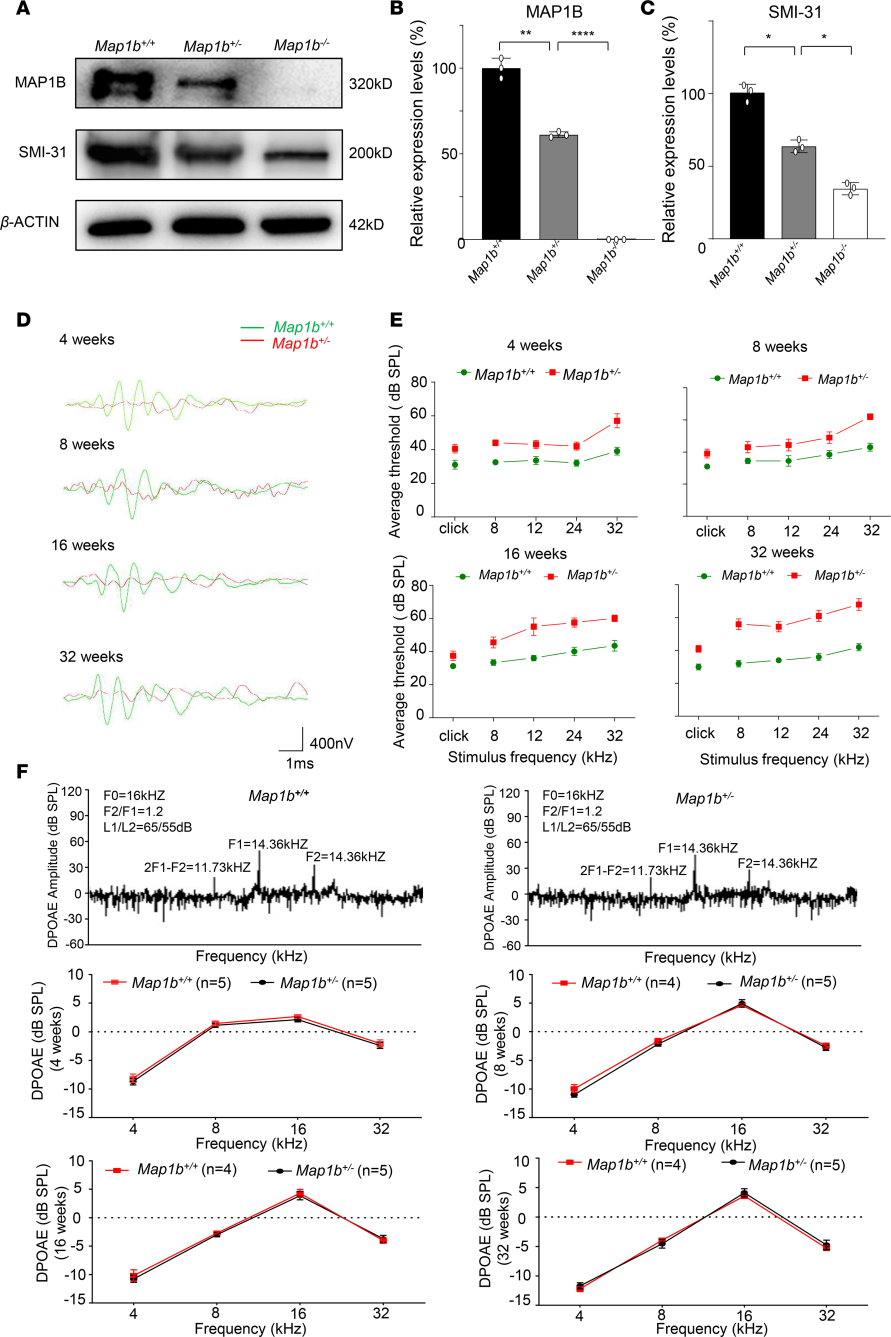
*Map1b*^+/–^ mice exhibited the hearing-impaired phenotype. (**A**) The expression of SMI-31 and MAP1B in the brain of *Map1b^–/–^*, *Map1b^+/–^*, and *Map1b^+/+^* mice at P7 using Western blot analysis. (**B** and **C**) Histogram quantifying the levels of SMI-31 and MAP1B in WT and *Map1b*-KO mice. Data are mean ± SEM of triplicates. (**D**) Representative ABR recordings evoked by click burst at 50 dB SPL of *Map1b*^+/+^ (green) and *Map1b*^+/–^ (red) mice at the age of 4, 8, 16, and 32 weeks. (**E**) Click (300–3000 Hz) and pure tone (8, 12, 24, 32 kHz) stimuli were presented to anesthetized mice. Significantly higher thresholds of ABR (dB SPL) were recorded in *Map1b^+/–^* mice, as compared with those of age-matched *Map1b^+/+^* ones (*n* = 10, including 5 male and 5 female animals). Data are mean ± SEM of triplicates. (**F**) DPOAE output of *Map1b*^+/–^ and WT mice (*n* = 10, including 5 males and 5 females) at 4, 8, 16, and 32 weeks old. Data are mean ± SEM of triplicates. *P <0.05, **P <0.01, and ****P <0.0001 by 1-way ANOVA followed unpaired Student’s *t* test in **B** and **C** and unpaired Student’s *t* test in **E** and **F**.

To investigate whether defects in *Map1b* caused the dysfunction of auditory system in vivo, we studied the *Map1b-*KO mice produced by the CRISPR/Cas9 system (Supplemental Figure 8). This deletion produced a frame-shift mutation resulting in a truncated Map1b protein with 133 amino acids (p.Leu139*). A Western blot analysis showed 40% decreases and absence of MAP1B protein in the brains of *Map1b*^+/–^ and *Map1b*^–/–^ mice at P7, respectively (Figure 7, A and B). All *Map1b^+/–^* mice were viable, while the *Map1b*^–/–^ mice only survived at 10 days after birth. To test if the deletion of *Map1b* altered the phosphorylation*,* we measured the levels of SMI-31 in the brain derived from *Map1b*^–/–^, *Map1b*^+/–^, and WT mice of P7. The levels of SMI-31 in *Map1b*^+/–^ and *Map1b*^–/–^ mice were 69% and 35% of WT littermates, respectively (Figure 7C). These data reveal that the deletion of *Map1b* caused the deficient phosphorylation of *MAP1B*. We concluded that the *Map1b^+/–^* mice recapitulated the biochemical phenotypes in the hearing-impaired patients.

To evaluate the effect of *Map1b* deletion on the hearing function, we measured the auditory brainstem response (ABR) to click and pure-tone-burst stimuli (8, 12, 24, and 32 kHz) in 4-, 8-, 16-, and 32-week-old *Map1b*^+/–^ and WT mice (38). As shown in Figure 7, D and E, ABR thresholds were significantly higher in the *Map1b*^+/–^ mice (5 males, 5 females) regardless of the age and sound frequency than in WT mice (5 males, 5 females). In particular, *Map1b*^+/–^ mice at the age of 4 weeks exhibited hearing impairment, evidenced by higher ABR thresholds than WT mice. Hearing impairment in mutant mice slowly progressed at the age of 8, 16, and 32 weeks, and these mice had elevated thresholds for all tested stimuli, as compared with WT mice. However, there were no significant difference of thresholds of ABR (decibel [dB] sound pressure level [SPL]) between male and female *Map1b^+/–^* mice (Supplemental Figure 9). We further assessed the hearing function by measuring DPOAE, which are used to estimate outer hair cell functions in the cochlea (39). A shown in Figure 7F, DPOAE (8, 12, 24, and 32 kHz) was not impaired in the 4-, 8-, 16-, or 32-week-old *Map1b*^+/–^ mice (5 males, 5 females), as compared with WT mice (5 males, 5 females). These data provide further evidence that *Map1b* deletion did not affect the functions of hair cells but may affect the function of spiral ganglia neurons.

**Figure 8:**
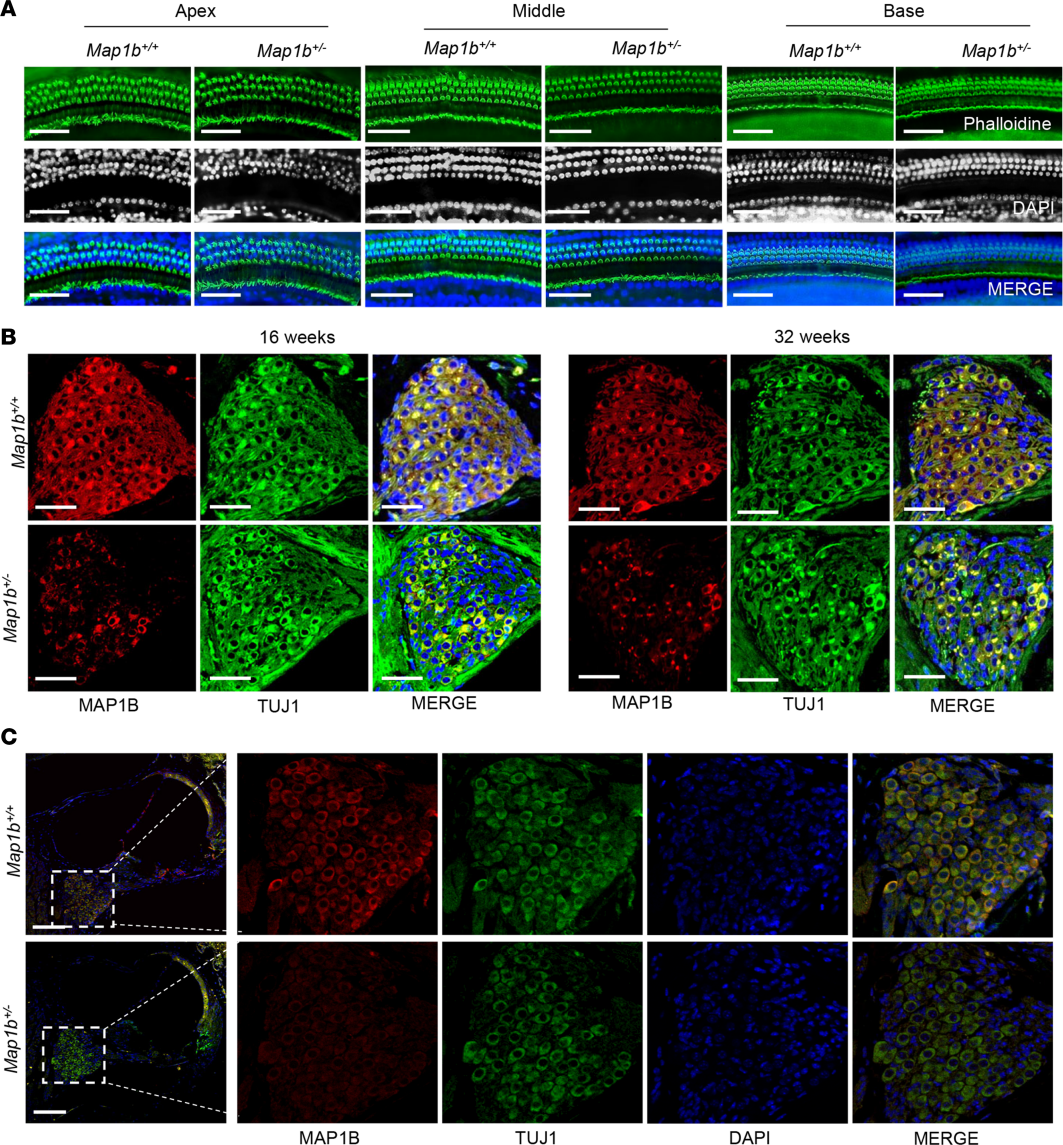
*Map1b* defect did not affect the structure and morphology of cochlea. (**A**) Representative images of inner ear hair cells in cochleae from *Map1b* heterozygous KO mice and WT littermates aged 4 weeks. Phalloidin was used to label the inner ear hair cells (green). Nuclei were stained with DAPI (blue). Scale bars: 50 μm. (**B**) Confocal images of MAP1B (red) and TUJ1 (green) double immunolabeling of SGNs in sectioned cochleae from 16- and 32-week-old mice. (**C**) Confocal images of MAP1B (red) and TUJ1 (green) double immunolabeling of SGNs in sectioned cochleae from 4 weeks mice. Scale bars: 100 μm. Three independent experiments were performed.

Subsequently, we examined the morphology of cochlea, including hair bundles and cuticular plate in the hair cells of mice cochlea, by labeling WT and *Map1b*^+/–^ mouse organs of Corti with fluorophore-conjugated phalloidin. As shown in Figure 8, the phalloidin staining revealed no apparent morphological abnormality or hair cell loss in middle turn, apex, and basal turns of cochlea in *Map1b*^+/–^ mice at the age of P30. This suggested that the loss of MAP1B may not affect the loss of hair and supporting cells of the organ of Corti. We then examined if the decreased expression of MAP1B affected the density of mice SGNs. As shown in Figure 8B, there were no substantial differences in the density of SGNs between WT and *Map1b*^+/–^ mice at various ages. However, the SGNs of *Map1b*^+/–^ mice at 4 weeks displayed pronounced decreases in the levels of MAP1B, as compared with those of WT mice (Figure 8C).

### MAP1B deficiency altered morphology and electrophysiology of SGNs.

**Figure 9:**
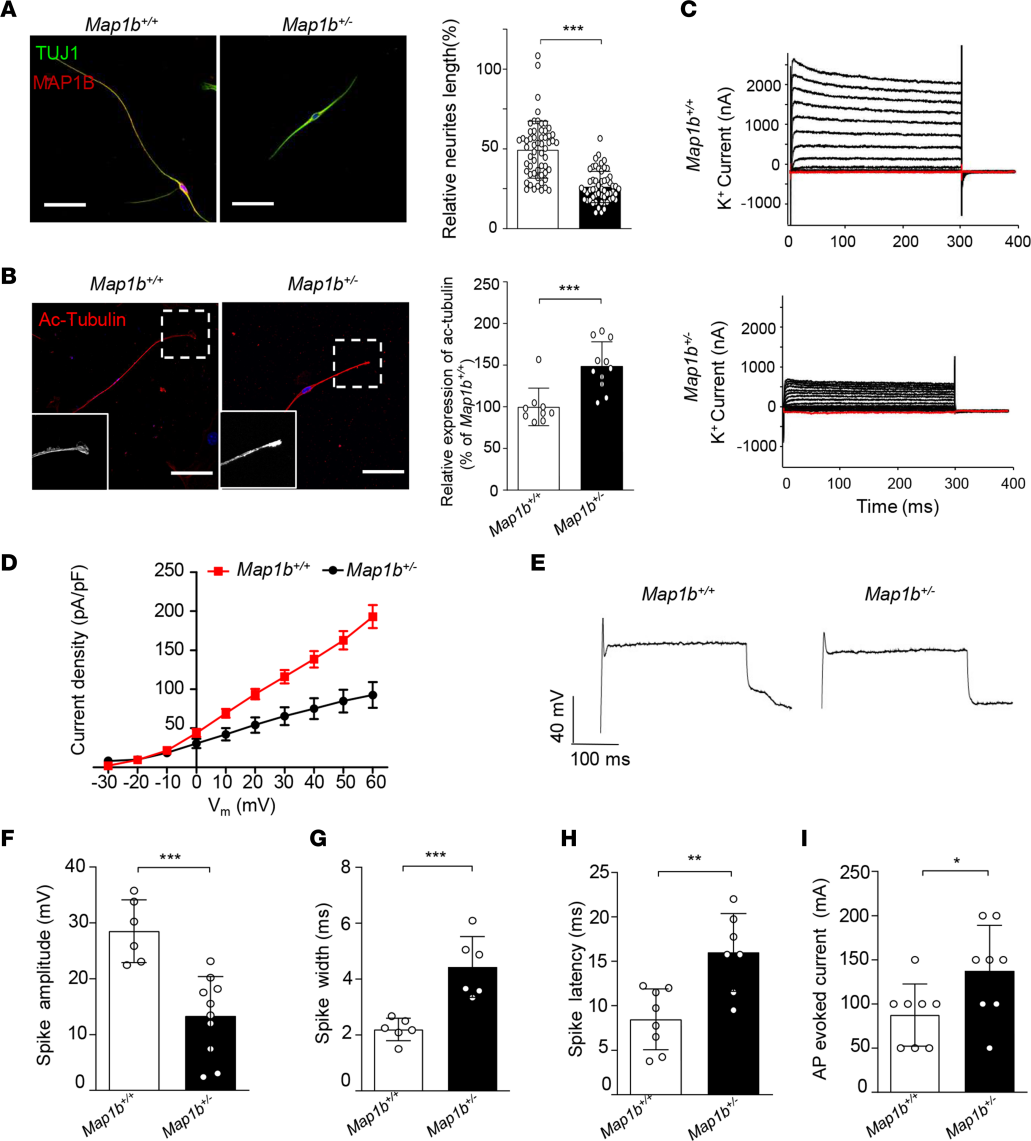
Morphology and electrophysiological properties in primary cultured mouse SGNs. (**A**) SGNs were explanted from *Map1b*^+/–^ and *Map1b*^–/–^ mice and stained with anti-TUJ1 (green). Scale bars: 50 μm. The lengths of neurites were summarized and normalized to the *Map1b*^+/+^ group. *n* = 57 and 58 for *Map1b*^+/–^ and *Map1b*^–/–^ SGNs, respectively. (**B**) Immunostaining images showing enriched level of acetylated tubulin (red) in *Map1b*^+/–^ SGNs. Scale bars: 50μm. Three independent experiments were performed. (**C**) Voltage-clamp recording of specific ion currents; example recording of delayed rectifier K^+^ current evoked by depolarizing steps ranging from –80 mV to 60 mV. (**D**) The average peak current densities in the *Map1b*^+/+^ and *Map1b*^+/–^ SGNs were statistically analyzed. (**E**) Example recording of a single action potential of both *Map1b*^+/+^ and *Map1b*^+/–^ SGNs. (**F**–**I**) Key differences between *Map1b*^+/+^ and *Map1b*^+/–^ SGNs are highlighted, including spike amplitudes (**F**), spike widths (**G**), spike latencies (**H**), and thresholds of action-potential activation (**I**). Data are mean ± SEM of triplicates. *P <0.05; **P <0.01; ***P <0.001 by unpaired Student’s *t* test.

We investigated whether MAP1B deficiency affected the morphology and function of SGNs, using the SGNs isolated from the cochlea of *Map1b*^+/–^ and *Map1b*^+/+^ mice of P5. First, we examined the neurite lengths in SGNs from *Map1b*^+/–^ and *Map1b^+/+^* mice. The loss of MAP1B shortened the neurite lengths in SGNs; the average lengths of neurites in neonatal *Map1b*^+/–^ mice were 126.9 ± 8.1 μm (*n* = 58), while those of *Map1b^+/+^* mice were 228.3 ± 10.7 μm (*n* = 57) (Figure 9A). Then, we evaluated whether the loss of Map1b altered the stability of microtubules by measuring the levels of acetylation of α-tubulin in SGNs. As shown in Figure 9B, evident increases in the acetylation levels of α-tubulin were observed in the growth cones of developing axons in *Map1b*^+/–^ SGNs, as compared with those of *Map1b*^+/+^ SGNs.

To assess whether the *Map1b* defects altered electrophysiological properties in SGNs, we performed whole cell voltage and current patch clamp recordings in isolated SGNs from *Map1b*^+/–^ and *Map1b^+/+^* mice of P5. In the voltage-clamp recording, the sustained portion of the outward current increased steadily with increasing depolarization (Figure 9C). The waveforms of the outward K^+^ currents in *Map1b*^+/–^ SGNs are distinct from those recorded from control littermates (Figure 9, C and D). Peak outward K^+^ current densities at all test potentials are significantly (*P* < 0.001) lower in *Map1b*^+/–^ SGNs than those in WT SGNs (Figure 9D). In particular, peak current densities at 60 mV in *Map1b*^+/–^ SGNs accounted for only 48% of those in WT SGNs. In the whole-cell current clamping recording, single action potentials in *Map1b*^+/–^ SGNs, induced by injected depolarizing currents, were different from those in *Map1b^+/+^* SGNs (Figure 9E). The SGNs from *Map1b*^+/–^ mice displayed significantly decreased spike amplitudes (~53.4%), prolonged spike width (~201.7%), longer spike latency (~122.5%), and raised threshold of action-potential activation (~157.1%), as compared with those of control littermates (Figure 9, F–I). These data demonstrate that *Map1b* deficiency altered the morphology and electrophysiology of SGNs in mice. We concluded that the *Map1b*^+/–^ mice recapitulated the phenotypes in hearing-impaired subjects carrying the MAP1B p.1400S>G mutation.

## Discussion

The pathophysiology underlying spiral ganglion cell defect–induced deafness remains elusive. Using the WES approach, in combination with functional assays and an animal disease model, we identified the potentially novel deafness-causative *MAP1B* gene encoding a highly conserved microtubule-associated protein. We demonstrated that the dysfunctions of SGNs caused by MAP1B deficiency led to hearing loss. Here, we show that 3 missense amino acid substitutions (p.1400S>G, p.923I>T, and p.1838F>L) of the *MAP1B* gene caused sensorineural hearing loss in the 3 genetically unrelated Chinese families with autosomal dominant inheritance of hearing loss. The MAP1B protein is a 2468–amino acid protein consisting of 2 microtubule-binding domains (MBD), 2 actin-binding domains (ABD), and a putative microtubule assembly helping domain (MTA) (17). The p.1400S>G, p.923I>T, and p.1838F>L mutations affected the functional domains of MAP1B protein (19). Particularly, the p.1400S>G mutation in the MTA domain of MAP1B affected the highly conserved phosphorylated site essential for the microtubule assemble (40, 41). In particular, the highly conserved Ser1400 in the MTA domain of MAP1B can be prime phosphorylated by DYRK1A, and it regulated the Ser1396 phosphorylation, which maintains a pool of dynamic microtubules (18). These mutations were only present in hearing-impaired members of these families at heterozygous form and not in 206 hearing normal subjects. Individuals bearing the *MAP1B* mutations developed milder to profound hearing loss at the average of 24 years, but not congenital conditions. Furthermore, no significant difference of DPOAE measurements between affected subjects and hearing-normal controls suggested that the hearing impairment may result from the altered functions in the spiral ganglia neurons (42). These mutations appeared to be the rare mutations, as only 1 proband carrying 1 of these mutations was detected in a cohort of 863 Chinese hearing-impaired subjects. The cosegregation of heterozygous mutations (p.1400S>G, p.923I>T, p.1838F>L) in the *MAP1B* gene with hearing impairment in these subjects of 3 Chinese pedigrees suggested that these *MAP1B* mutations are responsible for the development of hearing loss.

The higher expression of *Map1b* in the spiral ganglia neurons implicated its critical role in the hearing function. It was hypothesized that the substitution of Ser1400 with glycine by c.4198A>G mutation resulted in the deficient phosphorylation of MAP1B and, consequently, dysfunction of otic neurons. In this study, the OSN-like cells differentiated from those iPSCs derived from patient and control subjects allowed us to evaluate the effect of MAP1B p.1400S>G mutations on function of spiral ganglia neurons. Here, we demonstrated that the p.1400S>G mutation caused a neuronal differentiation defect in vitro. The primary defect of the p.1400S>G mutation was the deficient phosphorylation of MAP1B, evidenced by significantly decreased levels of SMI-31 (a marker for the type I phosphorylation) in the mutant OSN-like cells. In fact, this type I phosphorylation plays an important role in the microtubule stability and dynamics (40, 41). Here, the deficient phosphorylation of MAP1B was apparently responsible for the increasing acetylation of α-tubulin in the growth cones of otic neurons. The increased levels of acetylated-tubulin implied that the p.1400S>G mutation altered the stability and dynamics of microtubules in the OSN-like cells. Increasing evidences showed that microtubules are the critical components for the structure and function of neurons, especially in axons and dendrites of the synapses (43–45). Therefore, defects in microtubules affected the neuronal morphology, evidenced by shortening neurite lengths in mutant OSN-like cells bearing the p.1400S>G mutation. Strikingly, these mutant OSN-like cells exhibited defects in electrophysiological properties, including significantly decreased spike amplitudes, prolonged spike width, longer spike latency, and raised threshold of action-potential activation. Notably, these alterations in the morphology and function of the derived OSN-like cells were restored by genetic correction of *MAP1B* mutation using a CRISPR/Cas9-mediated gene editing. These data demonstrate that the MAP1B p.1400S>G mutation led to the OSN dysfunction necessary for the development of hearing loss.

We investigated biochemical and pathological consequences of Map1b defects in cochlea using the *Map1b*-KO mouse. The *Map1b*-KO mice recapitulated the biochemical and clinical phenotypes in hearing-impaired patients bearing the *MAP1B* mutation. In particular, markedly deficient phosphorylation of Map1b and substantial accumulation of acetylated α-tubulin observed in *Map1b*-KO mice were in a good agreement with those in OSN-like cells derived from patients carrying the p.1400S>G mutation. *Map1b*^+/–^ mice developed the hearing impairment phenotype at the age of 4 weeks and slowly progressed at the age of 8, 16, and 32 weeks. However, no impairment of DPOAE in the *Map1b*^+/–^ mice was comparable with those in the hearing-impaired patients bearing the *MAP1B* mutations. These data suggest that *Map1b* deletion did not affect the function of hair cells but may alter the function of spiral ganglia neurons. These were further supported by the observations that MAP1B deficiency did not affect the morphologies in middle turns, apex, and basal turns of cochlea of mice, in contrast with those in mice bearing other deafness-causing gene mutations such as CDC14A (46–48). Strikingly, MAP1B deficiency in the mice cochlea altered the morphology and function of SGNs rather than inner hair cells or outer hair cells in other deafness genes such as *LMO7* (48). This was supported by abnormal morphology of SGNs, including shortened neurite lengths, in the *Map1b*-KO mice. Furthermore, *Map1b* mutant mice displayed defects in the electrophysiological properties in the SGNs, as compared with those in the WT mice. As a result, the dysfunctions in the SGNs led to hearing impairment. Therefore, we concluded that the *Map1b*-KO mice recapitulated the biochemical and clinical phenotypes in the hearing-impaired subjects bearing the *MAP1B* mutation.

In conclusion, our study demonstrates that mutations in *MAP1B* gene encoding a microtubule-associated phosphoprotein caused defects in SGNs and subsequently caused sensorineural hearing loss. Our findings provide insights into pathophysiology of hearing loss arising from defects in SGNs and provide a step toward therapeutic interventions for this disorder.

## Methods

### Subjects and audiological evaluations.

DNA samples used for this investigation were from 30 members of 3 Chinese families carrying the *MAP1B* mutations and 861 genetically unrelated Chinese hearing-impaired subjects. The 206 control DNA samples were obtained from a panel of unaffected Chinese subjects from the same region. A comprehensive history and physical examination were performed to identify any syndromic findings, the history of the use of aminoglycosides, and genetic factors related to the hearing impairment in these subjects for this investigation. The audiological examination was performed, including pure-tone audiometry (PTA) and/or ABR, immittance testing, and DPOAE as detailed elsewhere (49, 50). The PTA was calculated from the sum of the audiometric thresholds at 500, 1000, 2000, 4000, and 8000 Hz. The severity of hearing impairment was classified into 5 grades: normal < 26 dB; mild = 26–40 dB; moderate = 41–70 dB; severe = 71–90 dB; and profound > 90 dB.

### WES.

WES of 2 hearing-impaired subjects (II-3 and II-4) of NB066 pedigree were performed by BGI as detailed elsewhere (51, 52). Genomic DNA was prepared according to the manufacturer’s instructions. High-quality genomic DNA was captured using the SureSelect XT Human All Exon 50 Mb kit (Agilent Technologies), and 90 bp paired-end sequencing was performed on a HiSeq 2000 instrument. Sequences were mapped to human genome assembly GRCh37.p10 at UCSC (Santa Cruz, California, USA). Software SOAPsnp was used to calculate a quality score for each consensus sequence and call genotypes in target regions. GATK (Indel Genotyper V1.0) was used to obtain capture and coverage statistics of exons and genomic positions. The threshold for filtering single-nucleotide polymorphisms (SNPs) included the following criteria: SNP quality score should be ≥ 20, sequencing depth should be between 4 and 200, estimated copy numbers should be no more than 2, and the distance between 2 SNPs should be larger than 5. SNPs from these analyses were summarized in Supplemental Table 1. Variants were annotated by ANNOVAR program. To further filter the SNP, the criteria for potential candidate variants included being nonsynonymous or in splice sites within 6 bps of an exon, having less than 1% mutant allele frequency in variant databases, and being cosegregated with the phenotype. The mutations were validated by Sanger sequencing in all family members and other genetically unrelated subjects.

### Sanger sequence analysis of MAP1B gene.

Three pairs of primers for PCR amplifying for genotyping for the c.4198A>G, c.2768T>C, and c.5512T>C mutations of *MAP1B* gene were used for this analysis. The forward and reverse primers for PCR amplification and sequence analysis are shown in Supplemental Table 4. DNA fragments of 30 members of 3 Han Chinese hearing-impaired families, 861 genetically unrelated Chinese hearing-impaired probands, and 206 control subjects were PCR amplified, purified, and subsequently analyzed by Sanger sequence. These sequence results were compared with the *MAP1B* genomic sequence (RefSeq NC_000005.10; https://www.ncbi.nlm.nih.gov/nuccore/NC_000005.10/).

### Generation of iPSCs from PBMNCs.

MNCs derived from hearing-impaired subject (NB066–II-4) and married-in control (NB066–II-6) were isolated from all blood via density gradient with Ficoll 400 (MilliporeSigma, F4375). Two million MNCs were transfected with 2 mg of plasmids each (pCXLE-EGFP, pCXLE-hSK, pCXLE-hUL, pCXLE-hOCT3/4-shp53-F, pCXWB-EBNA1; Addgene) using human CD34^+^ cell nucleofector kit (Lonza, VPA-1003) and then cultured in 12-well plates (defined as day 0) (53). On day 7, transfected cells were transferred to 6-well plates with prepared mouse embryonic fibroblast (MEF) feeder cells. Transfected cells were cultured in DMEM/F12 (Hyclone, SH30023.01B) supplemented with 20% KO serum replacement (Thermo Fisher Scientific, 10828-028), 1× GlutaMAX (Thermo Fisher Scientific, 35050-061), 1× nonessential amino acids solution (NEAA, Thermo Fisher Scientific, 11140050), 1× penicillin/streptomycin (Thermo Fisher Scientific, 15140122), 10 ng/mL basic fibroblast growth factor (bFGF, Peprotech, P09038), 100 μM β-mercaptoethanol (BME, MilliporeSigma, M3148), and 50 μg/mL ascorbic acid (MilliporeSigma, 1043003) and the medium was changed every other day. During day 3 to day 12, 0.5 mM sodium butyrate (MilliporeSigma, B5887) was added into culture medium. Colonies were manually picked after day 28.

### Genetic correction of iPSCs carrying the MAP1B p.1400S>G mutation.

Genetic correction of iPSCs carrying the p.1400S>G mutation was performed by CRISPR/Cas9 technology as detailed previously (54). The guide RNA sequence (sgRNA: 5′ - AAAGTTTTGTCTCCTTTACG - 3′) was designed in the CRISPR Design Tool and cloned into the plasmid pX459 (Addgene) to construct the plasmid pX459 sgRNA. Nucleofection technology was used to transfect the plasmid pX459-sgRNA and a 151 bp–corrected single-stranded donor oligonucleotide (ssODN) into mutant iPSCs by Human Stem Cell Nucleofector Kit (Lonza, VPH-5012). The cells were selected by 0.5 μg/mL puromycin 48 hours after nucleofection. Survival cells treated with 0.5 μg/mL puromycin were propagated into clones, collected, and analyzed by Sanger sequencing.

### Otic neuron differentiation from iPSCs.

iPSCs were induced toward otic neuron using a standard monolayer differentiation protocol with minor modifications (55). Briefly, iPSC colonies at 80% confluence were dissociated using Accutase (Thermo Fisher Scientific, A1110501) and pelleted at 1000 rpm for 5 minutes. Cells were then plated on Matrigel-coated 12-well plates and maintained in DMEM/F12 with 1× N2 (Stemcell Technologies, 07152), 1× B27 (Stemcell Technologies, 07100), 50 ng/mL FGF3 (R&D Systems, 1206), 50 ng/mL FGF10 (R&D Systems, 345-FG), and 500 ng/mL Noggin (Peprotech, 345-FG) for 10 days. To generate OSNs, the cells were dissociated and plated with Neurobasal Medium (Thermo Fisher Scientific, PHC1506) supplemented with 1× N2, 1× B27, 1× NEAA, and 1× GlutaMAX. The special media were added with 500 ng/mL of Sonic hedgehog (Shh-C24II, R&D Systems, 1845-SH) from day 3 and with 10 ng/mL of neurotrophin 3 (NTF3, R&D Systems, 267-GMP) and 10 ng/mL of BDNF (R&D Systems, 248-BDB) from day 7. To proliferate expansion, otic neuronal progenitors were cultured in a Neurobasal medium supplemented with 1× N2, 1× B27, 1× NEAA, 1× GlutaMAX, and 20ng/mL bFGF.

### Gene expression analysis.

Total cellular RNAs were extracted from various cells and tissues using TRIzol reagent (Invitrogen, 15596026) and reverse transcripted into cDNA using PrimeScript II 1st Strand cDNA Synthesis Kit (Takara, 6210A). qPCR was performed on the Applied Biosystems 7900HT Fast Real-Time PCR System. The data were analyzed using the 7900 System SDS RQ Manager Software, and relative gene expression was determined using the 2^–ΔΔCt^ method using β*-*actin as a housekeeping gene. Primer sequences for this study are listed in Supplemental Table 4.

### Western blot assays.

Western blot analysis was performed using 20 μg of total cellular proteins isolated from human cell lines or mice tissues, as detailed elsewhere (51, 56). The primary antibodies obtained from different companies were as follows: Proteintech (anti-MAP1B [21633-1-AP] and anti–ac-tubulin [Lys40; 66200-1-Ig]), Abcam (anti-BRN3A [ab81213], anti-TUJ1 [ab14545], anti-tubulin [ab15568], and anti–β-actin [ab8226]), and MilliporeSigma (anti–SMI-31 [NE1022]). Peroxidase Affinipure goat anti–mouse IgG and goat anti–rabbit IgG (Jackson ImmunoResearch Laboratories, West Grove, 111-005-146 and 111-005-144, respectively) were used as a secondary antibodies. Protein signals were visualized using the ECL system (CWBIO). Quantification of protein levels were determined by ImageJ software (NIH).

### Immunofluorescence staining and confocal microscopy.

Cells grown on matrigel-coated glass coverslips were fixed in 4% paraformaldehyde for 10 minutes, permeabilized with 0.5% Triton X-100 for 10 minutes, incubated with 5% BSA for 1 hour, and finally stained with primary antibodies at 4°C overnight. Subsequently, cells were washed with phosphate-buffered saline (PBS) and then incubated for 60 minutes with either Alexa Fluor 488– or Alexa Fluor 594–conjugated secondary antibodies (IgG, Invitrogen). Cells were then washed again in PBS and counterstained with DAPI solution and mounted with Dako fluorescence mounting medium. Cells were visualized and microphotographed using a FV1000 confocal laser-scanning microscope (Olympus).

### Generation of Map1b-KO mice.

CBA/CaJ mice were purchased from Zhejiang University Laboratory Animal Center. The *Map1b*-KO mice were generated using CRISPR/Cas9 approach. CRISPR/Cas9 genome editing in mice was performed as detailed previously (52, 57). Briefly, sgRNA target oligonucleotides (5′ - CGCTGCCCGCCATAAACTGC - 3′) were annealed and inserted into the pX330 vector (Addgene). The purified circular pX330 vector was microinjected into the pronucleus of the fertilized eggs and transferred into the oviducts of pseudopregnant CD1 female mice. For the genotypic analysis, total DNAs were extracted from the tails of pups, and a DNA fragment surrounding the target site in the exon 4 of *Map1b* was PCR amplified using specific primers (Supplemental Table 4) and confirmed by Sanger sequence.

### SGNs preparation and dissociation.

Early postnatal (P5–P7) CBA mice were dissected for primary cell harvesting for in vitro culture as detailed previously (58). Briefly, the mice were rapidly decapitated, the skull was opened, the brain was removed, and the temporal bones were transferred into a Petri dish containing sterile ice-cold PBS. Next, the membranous cochleae were dissected out of the temporal bones, followed by separation of the spiral ganglia and transfer to sterile ice-cold PBS. Spiral ganglia were pooled and cut into small pieces, and finally plated into 4-well plates with neurobasal medium containing 10 ng/mL brain-derived neurotrophic factor (BDNF, R&D Systems). The cultures were monitored daily under a microscope, and half of the culture medium was replaced with fresh medium every other day.

### Whole-cell patch-clamp recording.

For mouse SGNs and human differentiated cells, a whole-cell patch-clamp technique was applied using Axon MultiClamp 700B Microelectrode Amplifier (Molecular Devices) (59). Extracellular solution contained 150 mM NaCl, 5 mM KCl, 2 mM CaCl_2_, 1 mM MgCl_2_, and 10 mM glucose in 10 mM HEPES, pH 7.4. The intracellular (pipette) solution contained 130 mM K-gluconate, 5 mM KCl, 0.6 mM EGTA, 3 mM Na_2_ATP, 0.3 mM Na_2_GTP, and 10 mM C_4_H_10_N_3_O_5_P.2Na in 10 mM HEPES, pH 7.3. The data were acquired using the Axon Digidata 1550 analog-to-digital signal converter (Molecular Devices) and analyzed using the Clampfit software.

### Auditory brainstem response.

ABR recording experiment were performed as detailed elsewhere (38). ABR thresholds were recorded to assess the hearing of mice. Mutant and WT mice were anesthetized by i.p. injection of chloral hydrate (500 mg/kg) and the mouse temperature was maintained at 37°C–38°C. The evoked brainstem response to click (300–3000 Hz) and pure tones (8, 12, 24, 32 kHz) were amplified (50,000×) and averaged by 1024 times. The SPL from 90 to 10 dB in a 5 dB step and the lowest dB SPL level at which the ABR pattern could be recognized was defined as the ABR threshold. The Tucker-Davis Technologies (TDT) workstation provided the auditory stimulation, signal reception, and amplification; the coupled BioSigRZ software was used to analyze data.

### Statistics.

Statistical analysis was performed using GraphPad Prism (version 8.0.2) for statistical analysis to compare outcomes using a 2-tailed unpaired Student’s *t* test. For multiple comparisons, 1-way ANOVA was performed. *P* values of less than 0.05 were considered to be statistically significant.

### Study approval.

Informed consent in writing prior to their participation in this study were obtained from members of families and control subjects under protocols approved by the Ethic Committees of Zhejiang University School of Medicine. Furthermore, all experiments involving mice were approved by the IACUC of Zhejiang University School of Medicine.

## Author contributions

MXG and YC designed the experiments, monitored the project progression, data analysis, and interpretation. LC, JRC, QZ, and CC performed the generation of iPSC and OSN-like cells and biochemical analyses. JZ, HL, and YW performed the WES and mutational screening. LC, QH, HS, and SY carried out the mouse experiments. JZ, GP, SY, and RAF carried out the clinical evaluation. YC prepared the initial draft of the manuscript. RAF edited the manuscript and drafts. MXG made the final version of the manuscript.

## Supplementary Material

supplemental data

## Version 1 12/03/2020

Electronic publication
