# Supplementary material for: Mutations of MAP1B encoding a microtubule-associated phosphoprotein cause sensorineural hearing loss
Source: JCI Insight. 2020 Dec 3;5(23):e136046. doi: 10.1172/jci.insight.136046 (PMC7714412; doi:10.1172/jci.insight.136046)
Supplement: supplemental data [file jciinsight-5-136046-s069.pdf]

Supplemental data

Mutations of MAP1B encoding the microtubule-associated phosphoprotein cause sensorineural hearing loss

Limei Cui,[1,2,3] Jing Zheng,[1] Qiong Zhao,[1,2,3] Jia-Rong Chen,[1,2] Hanqing Liu,[2] Guanghua Peng,[4] Yue Wu,[1] Chao Chen,[1,2] Qiufen He,[2] Haosong Shi,[5] Shankai Yin,[5] Rick A. Friedman,[6] Ye Chen,[1,2,3] and Min-Xin Guan[1,2,3,6,7,8]

The supplemental data included the following information:

1. Supplemental Figure 1, 2, 3, 4, 5, 6, 7, 8 and 9
2. Supplemental Table 1, 2, 3 and 4

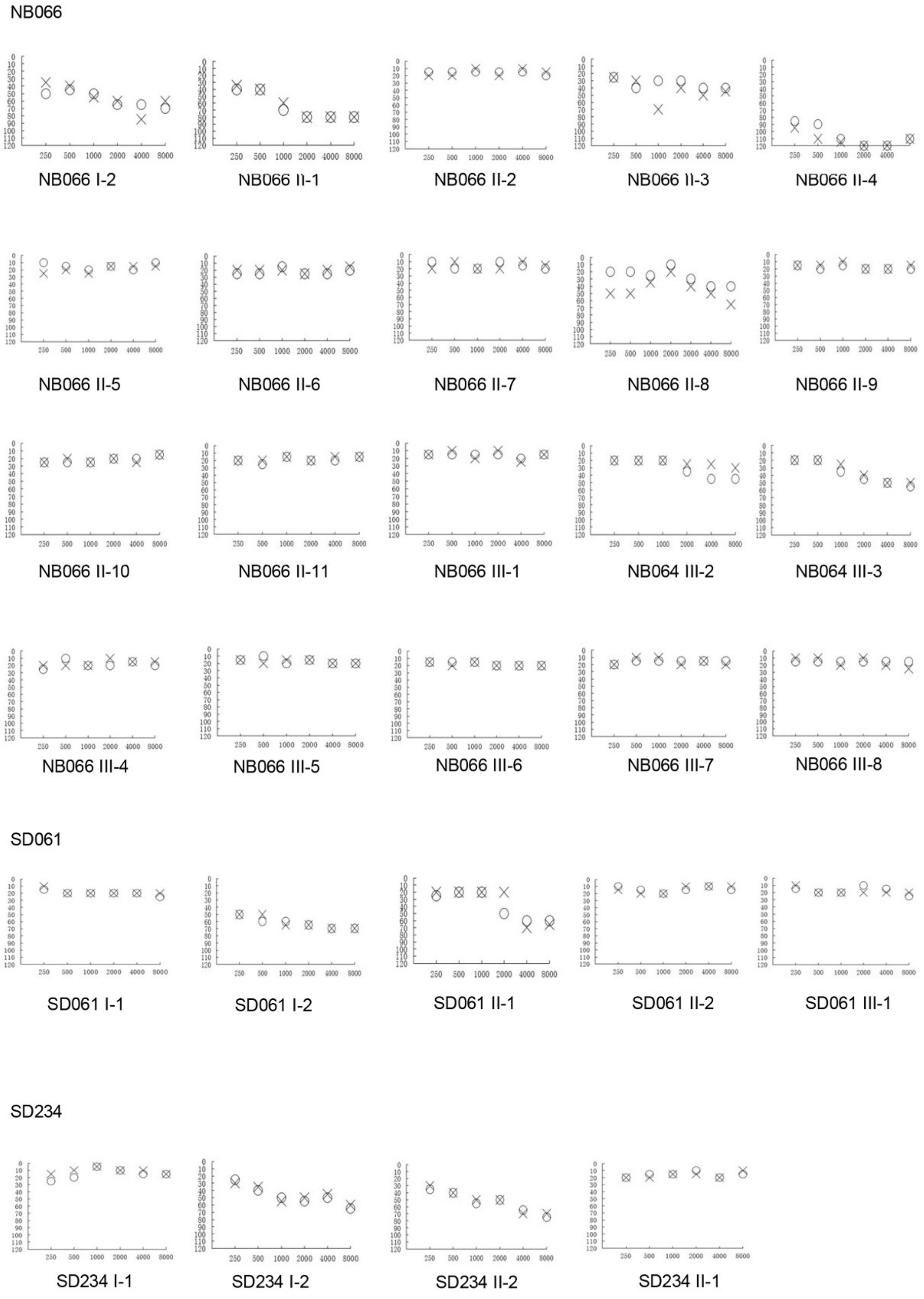

Supplemental Figure S1. Air conduction audiograms of the members of three Han Chinese families carrying *MAP1B* mutation(s). Symbols: X-left, O-right ear.

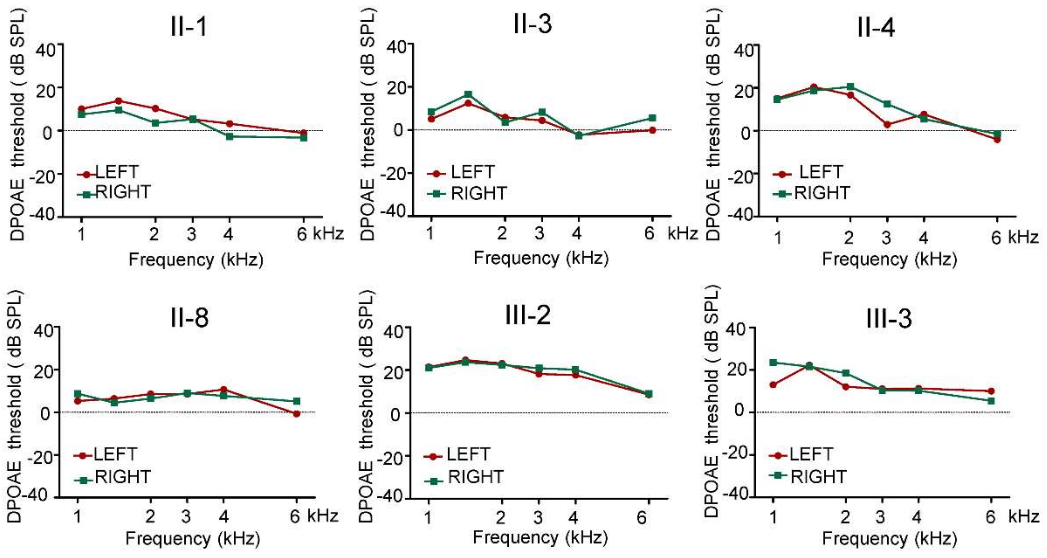

Supplemental Figure S2. DPOAE output of 6 members of the NB066 family.

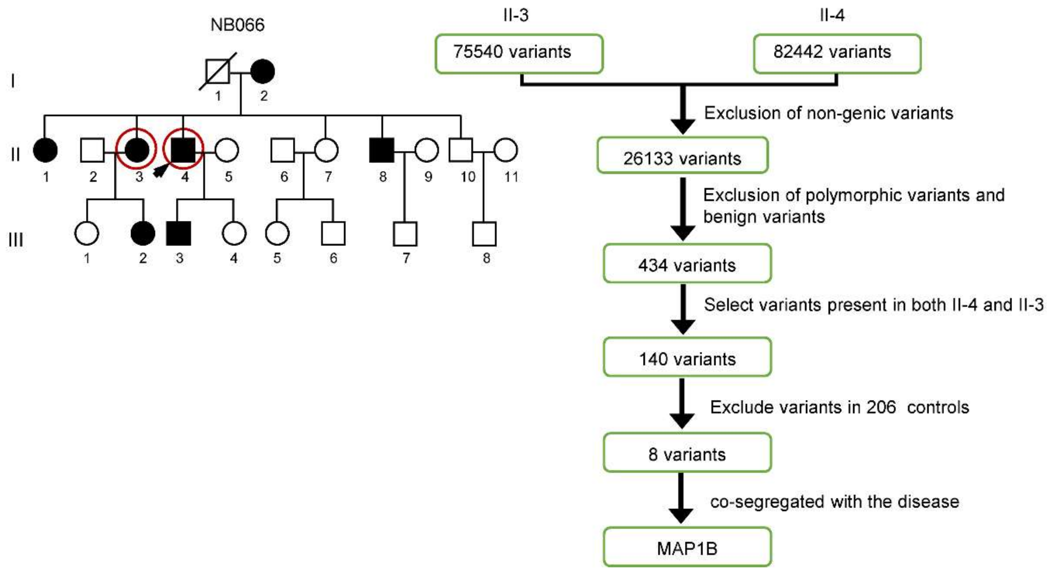

A B

Supplemental Figure S3. Identification of c.4198A>G (p.1400S>G) mutation in *MAP1B* gene. (A) Pedigree of the Chinese family (NB066). Hearing-impaired individuals were indicated by blackened symbols. (B) Summary of whole exome sequencing of two patients (NB066 II-3, NB066 II-4). The identified c.4198A>G (p.1400S>G) is located in the exon5 of *MAP1B*.

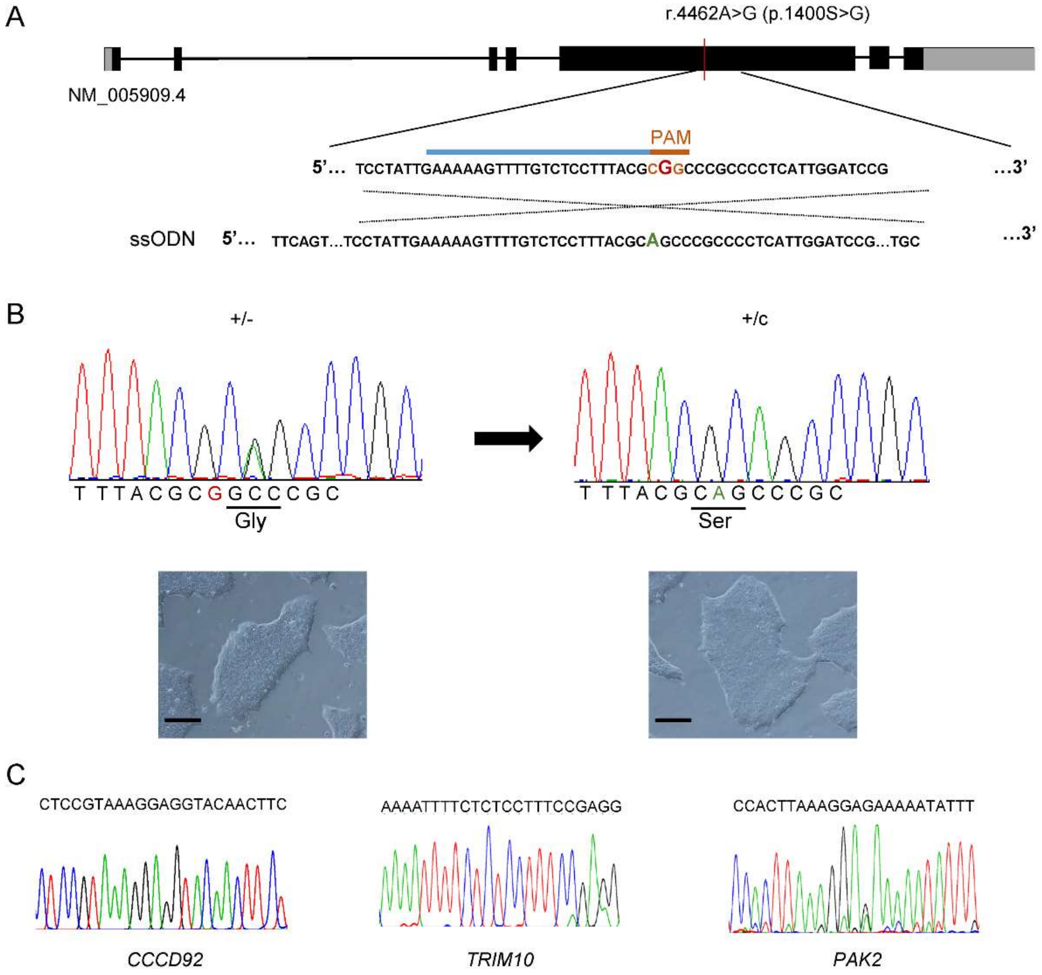

Supplemental Figure S4. Genetic correction of patient-derived iPSCs. (A) Schematic of the CRISPR-Cas9 strategy for genomic editing. (B) The positive clones of iPSCs were analyzed by Sanger sequencing to confirm the *MAP1B* mutation correction. Scale bars = 200 µm. (C) The top three scored off-target sites were also sequenced, and there was no double peaks adjacent to the off-target sites.

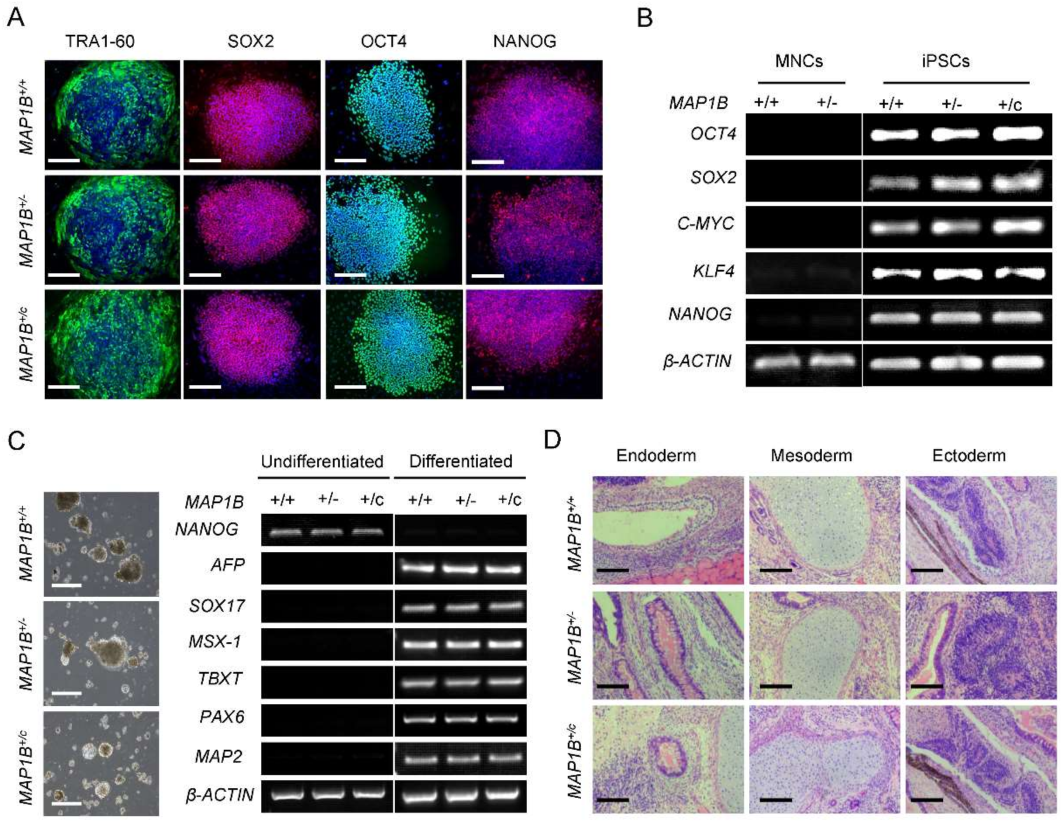

Supplemental Figure S5. Characterization of iPSCs from three different genotypes. (A) Confirmation of the differentiability of iPSCs by immunostaining for TRA1-60, SOX2, OCT4, and NANOG. Nuclei were stained with DAPI (blue). Scale bars = 100 μm. (B) Expression of iPSC-related markers at passage 4 (*OCT4*, *SOX2*, *c-MYC*, *KLF4*, and *NANOG*). (C) Formation of embryonic bodies at passage 4. Expression of related markers to confirm three germ-layered differentiation: *SOX17*, *AFP*, *SOX17*, *MSX-1*, *TBXT*, *PAX*, and *MAP2*. Scale bars = 400 μm. (D) Teratomas formed in NOD-SCID mice were sliced and stained with hematoxylin and eosin. Tissue structure characteristics of the three germ layers were observed (gut epithelium for endoderm, cartilage for mesoderm, as well as neural rosettes and retinal pigment epithelium for ectoderm). Scale bars = 100 μm.

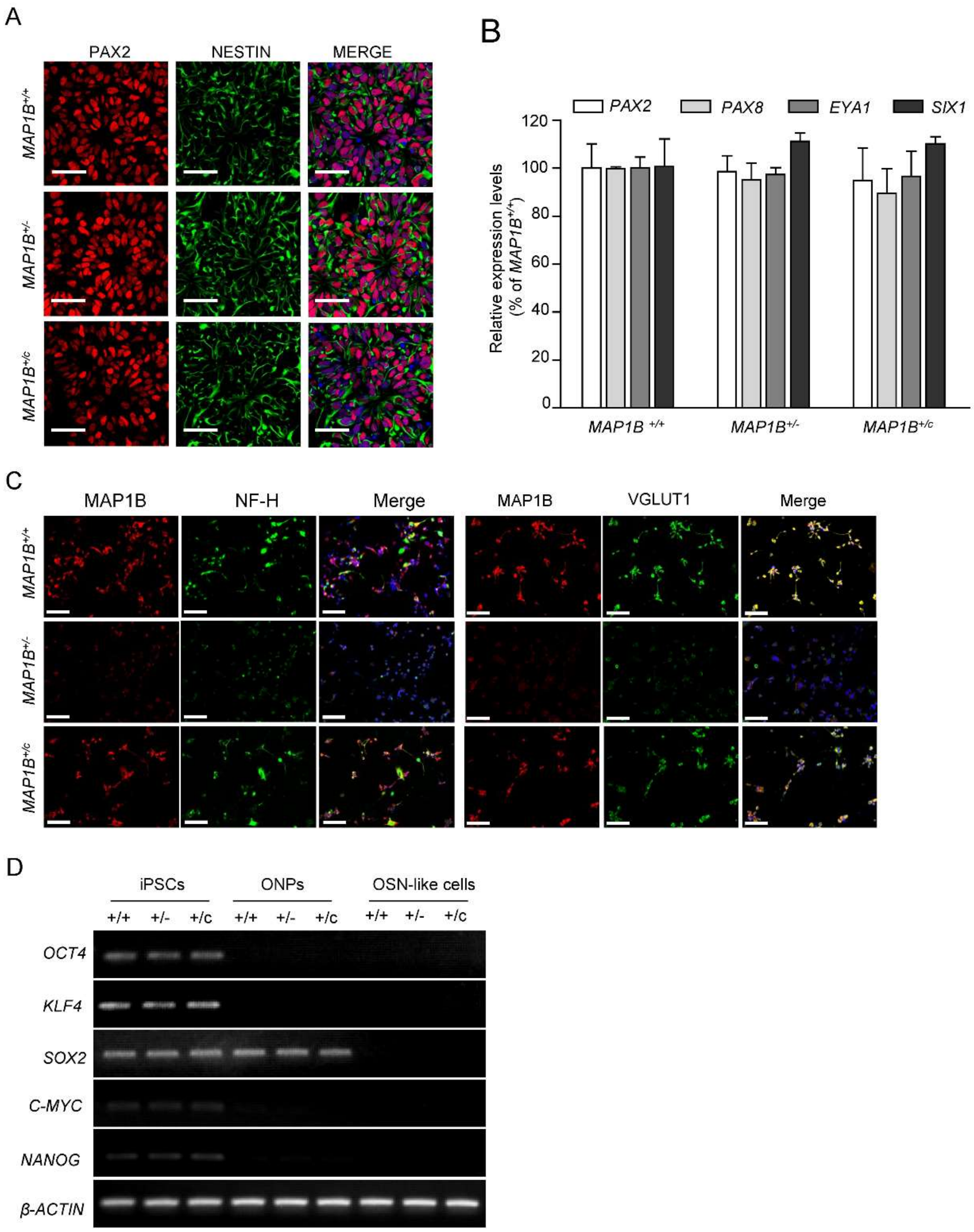

Supplemental Figure S6. Characterization of differentiated otic neuronal progenitor cells and otic sensory neuron-like cells. (A) iPSCs were induced toward otic neuronal progenitors and stained for the markers, PAX2+NESTIN. Nuclei were stained with DAPI (blue). Scale bars = 40 μm. (B) qRT-PCR analysis of the gene expression of otic neuronal progenitor markers. Marker genes, including *PAX2*, *PAX8*, *EYA1*, and *SIX1*, were detected in all three types of iPSCs induced toward the otic neurons. The relative gene expression were quantified and normalized to *β-ACTIN*. (C) Generation of otic neurons were confirmed by immunostaining with reported otic neuron marker NF-H and VGLUT1(green). Nuclei were stained with DAPI (blue). Scale bars = 50 μm. (D) The down regulation of pluripotency gene markers confirmed that majority of the cells had lost their pluripotency features by day 30 of differentiation.

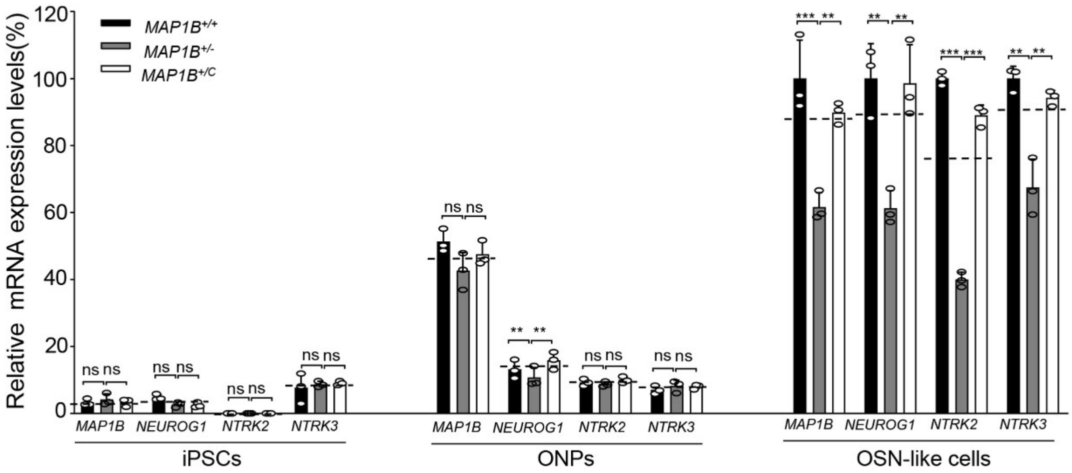

Supplemental Figure S7. qRT-PCR analysis of the gene expression of otic neuron markers in iPSCs, ONPs and OSN-like cells. The relative gene expression were quantified and normalized to *β-ACTIN*. Data are representative of three or more independent experiments. Error bars represent the SEM.

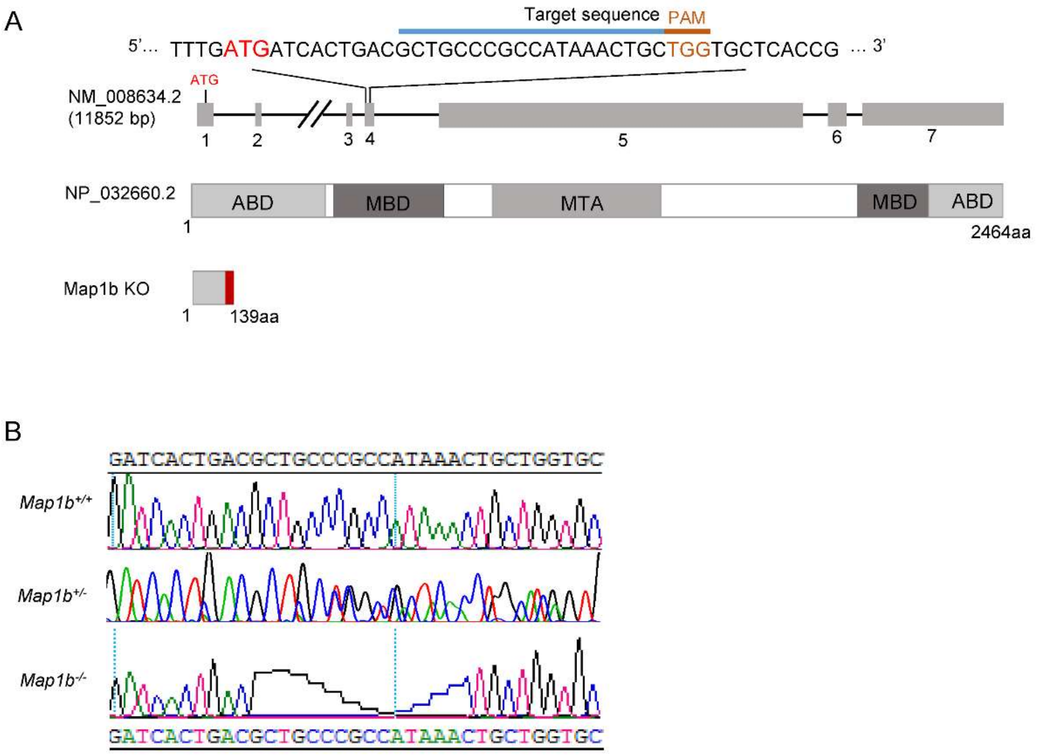

Supplemental Figure S8. Generation of *Map1b* KO mice using CRISPR/Cas9 technology. (A) Schematic diagram illustrating the processing of genetic knockout mice based on CRISPR/Cas9 technology. The gRNA sequence was targeted in exon 4, downstream of the second initiation codon. Frameshift mutation of *Map1b* knockout mice in codon 131 caused a premature stop codon in 139. (B) Partial sequence chromatograms of exon 4 of Map1b gene in WT, *Map1b*$^{+/-}$ and *Map1b*$^{-/-}$ mice revealed a 14bp deletion in *Map1b*.

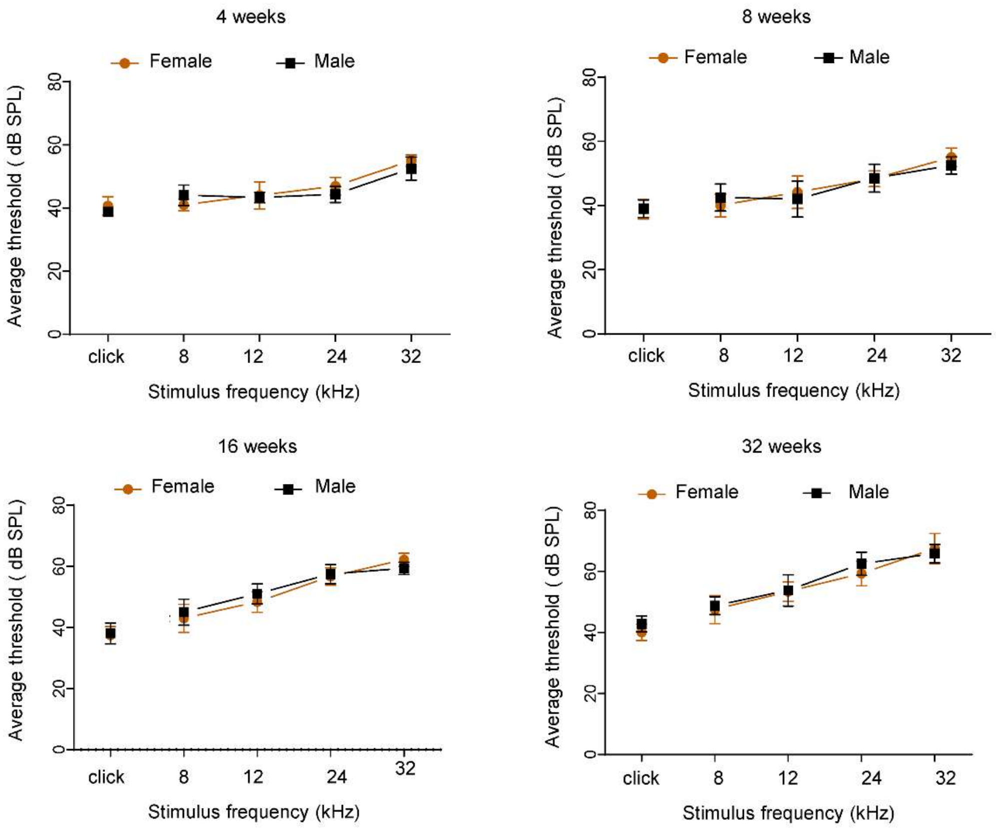

Supplemental Figure S9. ABR analysis of male and female *Map1b*$^{+/-}$ mice at the age of 4, 8, 16, and 32 weeks. Data are representative of three or more independent experiments. Error bars represent the SEM.

Supplemental Table S1. Summary of clinical data of members in three Chinese pedigrees

Subjects	Gender	Age at test/onset (years)	PTA(dB) right/left ear	Level of hearing impairment
NB066-I-2	F	87/56	58/56	Moderate
NB066-II-1	F	64/25	65/63	Moderate
NB066-II-2	M	66	16/16	Normal
NB066-II-3	F	61/30	34/48	Moderate
NB066-II-4	M	57/15	106/112	Profound
NB066-II-5	F	52	15/19	Normal
NB066-II-6	M	55	23/20	Normal
NB066-II-7	F	54	16/15	Normal
NB066-II-8	M	48/23	26/44	Moderate
NB066-II-9	F	45	18/16	Normal
NB066-II-10	M	47	22/22	Normal
NB066-II-11	F	46	19/18	Normal
NB066-III-1	F	40	16/16	Normal
NB066-III-2	F	36/18	31/23	Mild
NB066-III-3	M	35/22	38/34	Mild
NB066-III-4	F	33	18/16	Normal
NB066-III-5	F	32	17/18	Normal
NB066-III-6	M	24	18/19	Normal
NB066-III-7	M	25	16/16	Normal
NB066-III-8	M	28	15/16	Normal
SD061-I-1	M	65	20/20	Normal
SD061-I-2	F	62/20	63/62	Moderate
SD061-II-1	F	40/15	39/36	Mild
SD061-II-2	M	45	14/14	Normal
SD061-III-1	M	20	18/18	Normal
SD234-I-1	M	50	15/11	Normal
SD234-I-2	F	52/25	48/46	Mild
SD234-II-1	F	25	16/17	Normal
SD234-II-2	M	19/16	53/52	Moderate

Pure-tone audiometry: PTA; Decibel: dB.

Supplemental Table S2. Summary of exome sequencing data in two members of NB066 pedigree

Categories	II-3	II-4
Number of genomic positions for calling SNPs	134975362	135126064
Number of high-confidence genotypes	129513273	128088826
Number of high confidence genotypes in TR	50795761	50804878
Total number of SNPs	115027	111446
Synonymous –coding	11104	11041
Missense	10979	10955
Nonsense	107	113
Read through	48	45
Splice site	2757	2702
Intron	75966	73161
5’ UTR	3217	3099
3’ UTR	6387	6130
Intergenic	4462	4200
Hom	48487	47570
het	66540	63876
Frame error	0	0

Supplemental Table S3. Classification of three variants of *MAP1B* according to the ACMG guidelines.

Pos		NC_000005.9:g.71493380A>G	NC_000005.9:g.71491950T>C	NC_000005.9:g.71494694T>C
Nucleotide change		c.4198A>G	c.2768T>C	c.5512T>C
Amino acid change		p.1400S>G(Modified residue)	p.923I>T	p.1838F>L
dpSNP		rs753026898	rs143194383	rs139319889
frequency in general/ control population	GnomAD_exome_All	G=0.000068 (17/250672)	0.001347 (337/250230)	0.001786 (449/251420)
	GnomAD_exome_ EAS	G=0.00035(17/49002)	0.00120 (59/48972)	0.00247(121/49010)
	ExAC_ALL	0.000066 (8/120620)	0.001296 (156/120350)	0.001516 (184/121400)
	ExAC_ EAS	0.00032 (8/25148)	0.00128 (32/25090)	0.00270 (68/25166)
	Local	0	0	0
in silico predictions	SIFT	D	T	D
	Polyphen2- HVAR	B	B	B
	Mutation Taster	D	P(polymorphism)	P(polymorphism)
	LRT	D	N	N
	FATHMM	T	T	T
ACMG classification		Uncertain Significance (PM1+PM2+PP3+PP4)	Uncertain Significance (BP4)	Uncertain Significance (BP4)

PM1: mutational hot spot and/or critical, well established functional domain
PM2: with extremely low frequency in ExAC/GnomAD
PP3: in silico data support deleterious effect
PP4: phenotype match
BP4: in silico evidence support deleterious effect

Supplemental Table S4. List of primers used in the present study.

Primer names	Forward sequence (5`-3`)	Reverse sequence (5`-3`)
c.4198A>G mutation	ACAGCACCTTCGGAGATAATCCT	CGAGGTGACTGTGATAAAGACGATT
c.2768T>C mutation	CCTGCCAAAGAACTCGAAGC	TCCATGTCTTCTTCGGCTCG
c.5512T>C mutation	TATCCGATGTTGCTCCTCCCA	GTAGCCACTGTCACTTGGAGAT
OCT4	GACAGGGGGAGGGGAGGAGCTAGG	CTTCCCTCCAACCAGTTGCCCCAAAC
C-MYC	TGCACTGGAACTTACAACACCCGA	TAAGCAGCTGCAAGGAGAGCCTTT
KLF4	GAGGGAAGACCAGAATTCCCTTGA	AGAACCAAGACTCACCAAGCACCA
SOX2	GGGAAATGGGAGGGGTGCAAAAGAGG	TTGCGTGAGTGTGGATGGGATTGGTG
NANOG	TTTGTGGGCCTGAAGAAAACT	AGGGCTGTCCTGAATAAGCAG
AFP	GAATGCTGCAAACTGACCACGCTGGAAC	TGGCATTCAAGAGGGTTTTCAGTCTGGA
SOX17	GGTGTGAATCTCCCCGACAG	GGGGCAGGTCAAGCTTATGA
TBXT	GCCCTCTCCCTCCCCTCCACGCACAG	CGGCGCCGTTGCTCACAGACCACAGG
PAX6	ATGGGTGAGGGGTGTGTAGT	ATGGTGAAGCTGGGCATAGGCGGCAG
MSX-1	CGAGAGGACCCCGTGGATGCAGAG	GGCGGCCATCTTCAGCTTCTCCAG
MAP2	CAGGTGGCGGACGTGTGAAAATTGAGAGTG	CACGCTGGATCTGCCTGGGGACTGTG
PAX2	CACTTGCGAGCTGACACCTT	TGCAGATAGACTCGACTTGACTT
PAX8	AAGTGCAGCAACCATTCAACC	CTGCTCTGTGAGTCAATGCTTA
SIX1	GACTCCGGTTTTCGCCTTTG	TAGTTTGAGCTCCTGGCGTG
EYA1	TCAGATGCTATCTGCCGCTG	GTGCCATTGGGAGTCATGGA
RT-MAP1B	AAACGTCACTTCGGTGATGA	TGGGACACAAACCTGATTGA
NEUROD1	CTGTCCAGCTTGGAGGACC	GCCCCAGGGTTATGAGACTA
ISL1	ACGCATCACGAAGTCGTTC	CATGCTTTGTTAGGGATGGG
NTRK2	GAGATGTGATGGAGTGGGCT	CACTCCAAGTTTGGCATGAA
CCCDC92	GCAGGTGTCTGGAGTCTGTC	ATCCTACAAATGGGCTGCGT
TRIM10	GCAGGGGTTCCTTACGTTCT	CGGGTACAGAGGTGAGCAAG
PAK2	TGTTCCAACCTGGGCTTTGT	TCGATTTGCTATCGGCTGCT
β-*ACTIN*	CATGTACGTTGCTATCCAGGC	CTCCTTAATGTCACGCACGAT
Mice Map1b	GGGGACAGCTCGCTCTTACT	AAAGAGGCCCCTCCTACTACC
